# One-pot synthesis of enantiomerically pure *N*-protected allylic amines from *N*-protected α-amino esters

**DOI:** 10.3762/bjoc.12.94

**Published:** 2016-05-12

**Authors:** Gastón Silveira-Dorta, Sergio J Álvarez-Méndez, Víctor S Martín, José M Padrón

**Affiliations:** 1 Instituto Universitario de Bio-Orgánica “Antonio González” (IUBO-AG), Centro de Investigaciones Biomédicas de Canarias (CIBICAN), Universidad de La Laguna. C/ Astrofísico Francisco Sánchez 2, 38206, La Laguna, Spain

**Keywords:** amino acids, olefination, protecting group free, synthetic methods, Wittig reactions

## Abstract

An improved protocol for the synthesis of enantiomerically pure allylic amines is reported. *N*-Protected α-amino esters derived from natural amino acids were submitted to a one-pot tandem reduction–olefination process. The sequential reduction with DIBAL-H at −78 °C and subsequent in situ addition of organophosphorus reagents yielded the corresponding allylic amines without the need to isolate the intermediate aldehyde. This circumvents the problem of instability of the aldehydes. The method tolerates well both Wittig and Horner–Wadsworth–Emmons organophosphorus reagents. A better *Z*-(dia)stereoselectivity was observed when compared to the previous one-pot method. The (dia)stereoselectivity of the process was affected neither by the reaction solvent nor by the amount of DIBAL-H employed. The method is compatible with the presence of free hydroxy groups as shown with serine and threonine derivatives.

## Introduction

Allylic amines have received significant attention because they represent a common scaffold in diverse biologically relevant compounds and natural products [1]. In addition, allylic amines serve as versatile structural building block units for the synthesis of various functionalized organic compounds, playing an important role as intermediates in asymmetric synthesis [2]. Furthermore, asymmetric allylic amines can be obtained in enantiomerically pure form from conveniently functionalized commercially available amino acids [3–8]. Amino acids have been largely used in organic chemistry because they are widely accessible and they are relatively inexpensive, even on a bulk scale.

**Figure 1:**
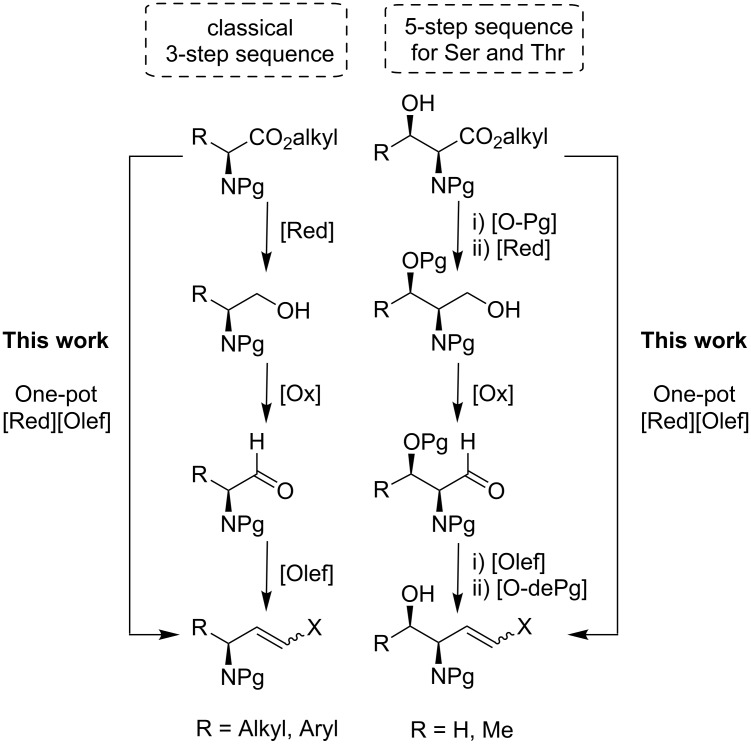
Strategies for the synthesis of *N*-protected allylic amines. [Red], reduction; [Ox], oxidation; [Olef], olefination; [Pg], protecting group.

The most classical and conventional protocol to obtain allylic amines from *N*-protected α-amino esters requires three chemical steps, i.e. reduction, oxidation and olefination (Figure 1) [9–10]. When the benzyl group is used as protecting group for the nitrogen functionality, the method represents a variation of the well-known Reetz protocol [11]. In the particular case of serine and threonine, additional *O*-protection and *O*-deprotection steps are required (Figure 1). Thus, Garner’s aldehyde [12–13] or *N*- and *O*-protected amino ester derivatives have been required [14].

All those methodologies involve long reaction sequences, including protection–deprotection and/or oxidation–reduction processes that are usually detrimental for the overall yield. Moreover, the classical three-step approach is highly undesirable in some cases not only because it generates more waste (more reactions are needed), but more importantly, because it implies the isolation of the aldehydes that could be unstable to manipulate.

As part of our interest in the synthesis of nitrogen-containing bioactive molecules [15], we developed a simplified version of the Reetz protocol [11] for the synthesis of enantiomerically pure *anti*-β-amino alcohols [16]. The process circumvents the problem of the instability of the aldehydes. It comprises the one-pot sequential reduction to aldehyde with DIBAL-H at −78 °C and subsequent in situ addition of Grignard reagents. Remarkably, our method is friendly with serine and threonine derivatives without the requisite to protect the β-hydroxy group. With this tool in hand, we thought on the possibility to extend the scope of the aforementioned one-pot procedure to the synthesis of enantiomerically pure allylic amines. It has been reported earlier the one-pot preparation of *N*-protected allylic amines via tandem DIBAL-H reduction–Wittig olefination of *N*-protected α-amino esters [17]. However, that preliminary study was limited to the use of phosphonium ylide reagents and commonly *t*-Boc (iBoc and Ac were used once) as *N*-protecting group. To the best of our knowledge, no further studies on the reaction conditions have been carried out. Instead, the method was applied to *N*-Ac aspartic, and *N*-Ac and *N*-Boc glutamic acid dialkyl esters, this time using a stabilized phosphonate ester [18]. This strategy was used later on in the synthesis of aminopeptidase A inhibitors [19]. Similarly, *N*-methylproline methyl ester was reacted in a similar one-pot fashion during the synthesis of nine-membered ring lactams [20]. Finally, a one-pot reduction-olefination involving an α-amino β-hydroxy ester and a phosphonium salt, both bearing free hydroxy groups, has been used in the synthesis of (−)-α-conhydrine [21].

In this work, our aim was to study in more detail this one-pot strategy. Similarly to our one-pot procedure to obtain enantiomerically pure *anti*-β-amino alcohols, we selected *N*,*N*-dibenzyl amino esters as starting material. We also investigated whether the method we developed could be applied to serine and threonine derivatives without protection of the hydroxy groups.

## Results and Discussion

The experimental procedure for the tandem reduction–Wittig olefination synthesis of allylic amines reported the use of toluene as solvent for the reduction step and THF as solvent to prepare the phosphonium ylide [17]. Consequently, the Wittig olefination takes place in a 2:1 (toluene/THF) solvent mixture. In the one-pot synthesis of *anti*-β-amino alcohols, the best results were achieved when the reactions were run in Et_2_O [16]. A fine tuning of the reducing agent was also necessary to obtain the desired products. Our first goal was to study the outcome of the reaction in terms of solvent and the amount of the reducing agent. When considering solvents for the process, we selected Et_2_O, THF and toluene. The initial amount of DIBAL-H was chosen on the basis of our one-pot procedure for the synthesis of *anti*-β-amino alcohols and set at 1.4 equiv.

As a model to study the reaction conditions we used as starting material (*S*)-methyl 2-(dibenzylamino)propanoate (**1**) [22]. In order to avoid the stereochemical drawback of the Wittig olefination (i.e., mixture of *E* and *Z* isomers), we selected the stabilized ylide ethyl 2-(triphenylphosphoranylidene)acetate, which gives the *E* isomer [23]. The results are summarized in Table 1. An excess of DIBAL-H had a negative impact in the yield leading to over reduction of the ester to the alcohol (Table 1, entries 1, 3 and 5). When considering the solvent, toluene gave the best results (Table 1, entry 6). In addition, only the *E* isomer of **2a** was obtained. The (dia)stereoselectivity (within limits of ^1^H NMR detection in the crude reaction mixture) of the process was affected neither by the reaction solvent nor by the amount of DIBAL-H employed. It is known from the literature that some racemization of enantiomerically pure aldehydes occurs during the DIBAL-H treatment. Although we have demonstrated earlier that no loss of enantiomeric purity was observed in the synthesis of *anti*-β-amino alcohols [16], we submitted commercially available DL-alanine to the aforementioned one-pot procedure to give *rac*-**2a**. Both *rac*-**2a** and **2a** were analyzed by chiral HPLC. The analysis confirmed that the enantiomeric purity was not affected by the process.

**Table 1:** Screening of the reaction conditions for the one-pot tandem reduction–olefination.

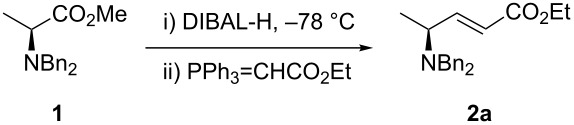				

Entry	Solvent	DIBAL-H (equiv)	*E*:*Z* ratio^a^	Yield (%)

1	Et_2_O	1.4	>20:1	5^b^
2	Et_2_O	1	>20:1	21
3	THF	1.4		–^b^
4	THF	1	>20:1	20
5	toluene	1.4		–^b^
6	toluene	1	>20:1	71
7	toluene	0.5	>20:1	40

^a^The *E*/*Z* ratio was determined by ^1^H NMR analysis of the crude product **2a**. ^b^Over reduction to alcohol.

The literature procedure reported the use of 2 equiv of DIBAL-H, whilst we found that only one equivalent is enough to avoid the excess of the reducing reagent. We hypothesized that compound **1** is half reduced by DIBAL-H forming the appropriate aluminoxy acetal, which is expected to be stable enough at temperatures as low as −78 °C. This is partly corroborated by the decomposition of the aluminoxy acetal to the over reduced alcohol when 1.4 equiv of DIBAL-H were used (Table 1, entries 1, 3 and 5) [24]. The presence of an aluminoxy acetal is also supported when THF is used, although the yields are low, probably due to the destabilization of the aluminoxy acetal (Table 1, entry 4) [25].

Once established the reaction conditions, we next studied the scope and limitations of the one-pot protocol using a small subset of assorted Wittig and Horner–Wadsworth–Emmons (HWE) organophosphorus reagents (Table 2). When considering phosphonium ylides (Table 2, entries 1 and 2), better *Z*-(dia)stereoselectivity was observed when compared to the previous one-pot method. The olefination with the semistable ylide obtained by the addition of KN(TMS)_2_ to benzyltriphenylphosphonium bromide yielded **2b** as an inseparable mixture of *E* and *Z* isomers in almost equal amounts. This result represents an improvement in selectivity toward the *Z* isomer when compared to the 5–7:1 *E*:*Z* ratio reported [17]. The preparation of (*E*)-**2b** has been reported earlier [6] but this is the first time it is described the synthesis of its (dia)stereoisomer (*Z*)-**2b**. The olefination with the non-stabilized ylide of pentadecyltriphenylphosphonium bromide led exclusively to (*Z*)-**2c**.

**Table 2:** Influence of the organophosphorus reagent in the outcome of the one-pot tandem reduction–olefination.

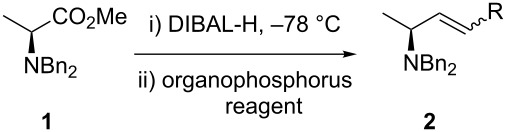				

Entry	Organophosphorus reagent	Product	*E*:*Z* ratio^a^	Yield (%)

1	Ph_3_P=CHPh	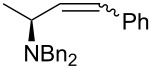 **2b**	1:1.3	60
2	Ph_3_P=CH(CH_2_)_13_Me	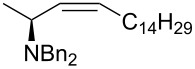 **2c**	>1:20	40
3	Ph_3_P=CHCN	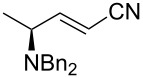 **2d**	5:1	72
4	Ph_3_P=C(Me)CO_2_Et	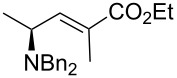 **2e**	>20:1	71
5	(MeO)_2_P(O)CH_2_CO_2_Me	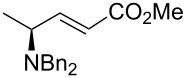 **2f**	>20:1	68
6	(CF_3_CH_2_O)_2_P(O)CH_2_CO_2_Et	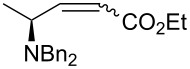 **2g**	1:1.6	78

^a^The *E*/*Z* ratio was determined by ^1^H NMR analysis of the crude product.

Likewise, the results obtained for (*E*)-**2a**, the use of stabilized ylides (Table 2, entries 3 and 4) provided the expected *E* alkenes. In the particular case of the α-cyano phosphorane reagent, complete stereoselectivity was not achieved (*E*:*Z* ratio of 5:1) as it has been previously observed in similar reactions [26]. Fortunately, both products were easily separated by column chromatography.

Finally, HWE reagent led to the preparation of (*E*)-**2f** as sole isomer in 68% yield (Table 2, entry 5), whilst the Still–Gennari variant [27] gave the mixture of (*E*)-**2g** and (*Z*)-**2g** isomers (Table 2, entry 6). At this point, we wondered if our methodology for the synthesis of allylic amines was compatible also with free hydroxy groups present in the substrate, as it has been shown earlier for a *N*-Boc protected amino hydroxy ester [21]. To corroborate this idea, *N*,*N*-dibenzylamino benzyl ester of L-serine (**3**) was submitted to the one-pot tandem reduction–olefination procedure described above. Disappointingly, the product (*E*)-**4a** was obtained in low yield although in excellent (dia)stereoselectivity. In the synthesis of *anti*-2-amino-1,3-diols, we reported earlier that the addition of DIBAL-H must be done necessarily in two portions [16]. Thus, fine-tuning of the reduction conditions was required in order to improve the yield, as shown in Table 3.

**Table 3:** Screening of the reaction conditions for the one-pot tandem reduction–olefination of free hydroxyserine derivatives.

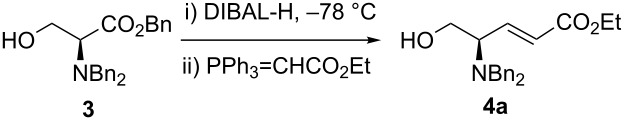				

Entry	Reduction time (h)	DIBAL-H (equiv)	*E*:*Z* ratio^a^	Yield (%)

1	2	1	>20:1	7
2	2	1.4	>20:1	36
3	2	2	>20:1	58
4	i) 1 ii) 2	i) 1.4^b^ ii) 0.5	>20:1	65

^a^The *E*/*Z* ratio was determined by ^1^H NMR analysis of the crude product. ^b^DIBAL-H was added in two portions.

The best results were obtained when the reducing agent was added in two portions, with an interval of one hour between the two (Table 3, entry 4). It should be taken into account that the DIBAL-H addition must be done necessarily in two portions (Table 3, entry 3 vs 4) in order to increase the yield of the reaction. We speculate that the free hydroxy group coordinates to the DIBAL-H, making compulsory the additional amount of reducing agent.

As shown in Table 4, this modification of the one-pot protocol is compatible with the use of HWE organophosphorus reagents (Table 4, entry 1). However, the reaction with the non-stabilized ylide of pentadecyltriphenylphosphonium bromide resulted in loss of stereoselectivity (Table 4, entry 2). The method can be applied also to the *N*,*N*-dibenzylamino benzyl ester of L-threonine (**5**) albeit lower yields are obtained. It is remarkable that this protocol avoids using *O*-protecting groups or the Garner aldehyde which have been used extensively in the synthesis of related compounds.

**Table 4:** One-pot tandem reduction–olefination of free hydroxyserine and threonine derivatives.

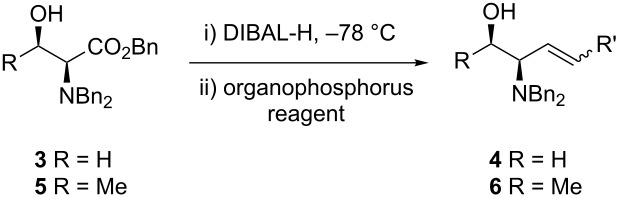				

Entry	Organophosphorus reagent	Product	*E*:*Z* ratio^a^	Yield (%)

1	(MeO)_2_P(O)CH_2_CO_2_Me	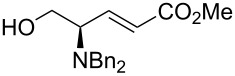 **4b**	>20:1	50
2	Ph_3_P=CH(CH_2_)_13_Me	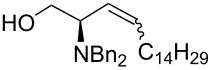 **4c**	37:63	60^b^
3	Ph_3_P=CHCO_2_Et	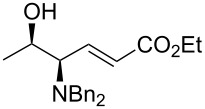 **6a**	>20:1	40
4	Ph_3_P=C(Me)CO_2_Et	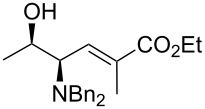 **6b**	>20:1	39

^a^The *E*/*Z* ratio was determined by ^1^H NMR analysis of the crude product. ^b^Inseparable *E*/*Z* mixture.

## Conclusion

In summary, this simple protocol described herein enables a rapid access to a number of useful enantiopure allylic amines from readily available amino acids. Optically active amino ester derivatives can be transformed into allylic amines by a tandem reduction–olefination procedure. The process avoids the isolation of the intermediate aldehyde, which makes it an attractive option for unstable aldehydes. The selectivity in the olefination step was considerably higher than those previously reported using batch methods with related reagents. Our technique is also compatible with free hydroxy groups displayed in the substrate, allowing the synthesis of new products unreported to date. Further investigations into the reaction mechanism, scope, and application of this strategy are currently underway in our laboratory.

## Supporting Information

File 1 General procedures, analytical data and spectra of all compounds, methods for conversion.
