# Supplementary material for: One-pot synthesis of enantiomerically pure N-protected allylic amines from N-protected α-amino esters
Source: Beilstein J Org Chem. 2016 May 12;12:957–62. doi: 10.3762/bjoc.12.94 (PMC4902051; doi:10.3762/bjoc.12.94)
Supplement: File 1 — General procedures, analytical data and spectra of all compounds, methods for conversion. [file Beilstein_J_Org_Chem-12-957-s001.pdf]

Supporting Information

for

One-pot synthesis of enantiomerically pure *N*-protected allylic amines from *N*-protected α-amino esters

Gastón Silveira-Dorta, Sergio J. Álvarez-Méndez, Víctor S. Martín, José M. Padrón*

Address: Instituto Universitario de Bio-Orgánica “Antonio González” (IUBO-AG), Centro de Investigaciones Biomédicas de Canarias (CIBICAN), Universidad de La Laguna. C/ Astrofísico Francisco Sánchez 2, 38206, La Laguna, Spain

Email: José M. Padrón - jmpadron@ull.es

*Corresponding author

General procedures, analytical data and spectra of all compounds, methods for conversion

Table of Contents

General S3

General procedures S3

Compound characterization data S5

Figure S1: ^{1}H and ^{13}C NMR spectra for compound **1** S9

Figure S2: ^{1}H and ^{13}C NMR spectra for compound **2a** S10

Figure S3: ^{1}H and ^{13}C NMR spectra for compound **2b** S11

Figure S4: ^{1}H and ^{13}C NMR spectra for compound **2c** S12

Figure S5: ^{1}H and ^{13}C NMR spectra for compound (*E*)-**2d** S13

Figure S6: ^{1}H and ^{13}C NMR spectra for compound (*Z*)-**2d** S14

Figure S7: ^{1}H and ^{13}C NMR spectra for compound **2e** S15

Figure S8: ^{1}H and ^{13}C NMR spectra for compound **2f** S16

Figure S9: ^{1}H and ^{13}C NMR spectra for compound **2g** S17

Figure S10: ^{1}H and ^{13}C NMR spectra for compound **3** S18

Figure S11: ^{1}H and ^{13}C NMR spectra for compound **4a** S19

Figure S12: ^{1}H and ^{13}C NMR spectra for compound **4b** S20

Figure S13: ^{1}H and ^{13}C NMR spectra for compound **4c** S21

Figure S14: ^{1}H and ^{13}C NMR spectra for compound **5** S22

Figure S15: ^{1}H and ^{13}C NMR spectra for compound **6a** S23

Figure S16: ^{1}H and ^{13}C NMR spectra for compound **6b** S24

Figure S17: HPLC analysis of *rac*-**2a** and **2a** S25

Table S1: ^{1}H NMR data comparison with literature data for **2a** S27

Table S2: ^{13}C NMR data comparison with literature data for **2a** S27

Table S3: ^{1}H NMR data comparison with literature data for **2b** S28

Table S4: ^{13}C NMR data comparison with literature data for **2b** S28

Table S5: ^{1}H NMR data comparison with literature data for (*E*)-**2d** S29

Table S6: ^{13}C NMR data comparison with literature data for (*E*)-**2d** S29

Table S7: ^{1}H NMR data comparison with literature data for (*Z*)-**2d** S30

Table S8: ^{13}C NMR data comparison with literature data for (*Z*)-**2d** S30

Table S9: ^{1}H NMR data comparison with literature data for **4c** S31

Table S10: ^{13}C NMR data comparison with literature data for **4c** S32

References S33

General

^{1}H NMR spectra were recorded at 400 and 500 MHz at 298 K, ^{13}C NMR spectra were recorded at 100 and 125 MHz, respectively. Chemical shifts were reported in units (ppm) by assigning TMS resonance in the ^{1}H NMR spectrum as 0.00 ppm ($CDCl_3$, 7.26 ppm). Data were reported as follows: chemical shift, multiplicity (s = singlet, d = doublet, t = triplet, q = quartet, quin = quintuplet, sex = sextet, dd = double doublet, ddd = double double doublet, m = multiplet and br = broad), coupling constant (*J* values) in Hz and integration. Chemical shifts for ^{13}C NMR spectra were recorded in ppm from tetramethylsilane as the internal standard using the central peak of $CDCl_3$ (77.0 ppm). Reagent-grade chemicals were obtained from diverse commercial suppliers and were used as received. A freshly opened bottle of diisobutylaluminium hydride was used. Optical rotations were measured with a polarimeter at the sodium line at different temperatures in $CHCl_3$. Accurate masses (HRMS) were determined by electrospray ionization (ESI-TOF) and electron impact (EI-TOF). Reactions were monitored using thin-layer chromatography (TLC) on aluminum packed percolated Silica Gel 60 F_{254} plates. Flash column chromatography was carried out with silica gel 60 (particle size less than 0.020 mm) by using appropriate mixtures of ethyl acetate and hexanes, or diethyl ether and hexanes as eluents. Compounds were visualized by use of UV light and 2.5% phosphomolybdic acid in ethanol. Reactions were performed using oven-dried glassware. All reactions involving air- or moisture-sensitive materials were carried out under argon atmosphere. Anhydrous magnesium sulfate was used for drying solutions. Chemical nomenclature was generated using ChemBioDraw Ultra 13.0.

General procedures

General procedure A: preparation of N-protected allylic amines

To a solution of (*S*)-methyl 2-(dibenzylamino)propanoate (**1**, 1 mmol) in dry toluene (3 mL) was added dropwise DIBAL-H (1.0 M solution in hexanes, 1 mmol) at −78 °C. After stirring for 2 h at −78 °C, the appropriate organophosphorus reagent was added in small portions (or dropwise) at −78 °C (some of them required to be prepared separately, see below). The mixture was allowed to warm gradually to 0 °C. Then, the mixture was quenched with saturated Rochelle's salt solution (10 mL). The reaction mixture was vigorously stirred for 2 h at rt. After dilution with water (10 mL), the biphasic mixture was separated and extracted with Et_2O (3 × 10 mL). The combined organic phases were washed with brine, dried over $MgSO_4$, filtered and concentrated in vacuum. The residue was purified by flash chromatography on silica gel (eluent EtOAc/hexanes) to give the corresponding *N*-protected allylic amine.

General procedure B: preparation of N-protected β-hydroxy allylic amines

To a solution of (*S*)-benzyl 2-(dibenzylamino)-3-hydroxypropanoate (**3**) or (2*S*,3*R*)-ethyl 2-(dibenzylamino)-3-hydroxybutanoate (**5**) (0.27 mmol) in dry toluene (3 mL) was added DIBAL-H in two portions, first dropwise at −78 °C (0.41 mL, 1.0 M solution in hexanes, 0.41 mmol) and the rest 1 h later (0.14 mL, 1.0 M solution in hexanes, 0.14 mmol). After stirring for 2 h at −78 °C, the appropriate organophosphorus reagent was carefully added at –78 °C (some of them were prepared separately, see below). The mixture was allowed to warm gradually to 0 °C. Then, the mixture was quenched with saturated Rochelle's salt solution (10 mL). The reaction mixture was vigorously stirred for 2 h at rt. After dilution with water (10 mL), the biphasic mixture was separated and extracted with Et_2O (3 × 10 mL). The combined organic phases were washed with brine, dried over $MgSO_4$, filtered and concentrated in vacuum. The residue was purified by flash chromatography on silica gel (eluent EtOAc/hexanes) to give the corresponding *N*-protected β-hydroxy allylic amine.

General procedure C: preparation of Wittig organophosphorus reagents

To a stirred suspension of the appropriate phosphonium bromide (1.0 mmol) in dry toluene (10 mL) was added dropwise $KN(TMS)_2$ (1 mL, 0.5 M solution in toluene, 1.0 mmol) at 0 °C. After 30 min the flask was cooled to -78 °C.

General procedure D: preparation of HWE organophosphorus reagents

To a solution of the appropriate phosphonate (0.5 mmol) in dry toluene (2 mL) at 0 °C, was added carefully NaH (60% in mineral oil, 0.5 mmol). The mixture was stirred for 4 h at 0 °C.

General procedure E: preparation of Still–Gennari organophosphorus reagents

To freshly distilled THF (20 mL) was added $KN(TMS)_2$ (2 mL, 0.5 M in THF, 1 mmol) and 18-crown-6 (280 mg, 1.1 mmol). The reaction mixture was cooled to −78 °C and a solution of the appropriate phosphonate (1.0 mmol) in 10 mL of dry THF was added via cannula. The reaction was stirred 30 min at −78 °C and then 1 h at 0 °C. Then, the mixture was cooled to −78 °C.

Compound characterization data

*(S,E)-Ethyl 4-(dibenzylamino)pent-2-enoate (**2a**)* [1]

The general procedure A was applied to **1** on a 0.35 mmol (100 mg) scale using ethyl 2-(triphenylphosphoranylidene)acetate (261 mg, 0.51 mmol), to give after purification (eluent Et_2O/hexanes 15:85) **2a** (80.3 mg, 71%, *E/Z* >20:1) as a colorless oil. $[\alpha]^{25}_D$ = -136.0 (*c*, 1.00, $CHCl_3$).

*(S,Z/E)-N,N-Dibenzyl-4-phenylbut-3-en-2-amine (**2b**)* [2]

The general procedure A was applied to **1** on a 0.35 mmol (100 mg) scale using the ylide of benzyltriphenylphosphonium bromide (303.3 g, 0.7 mmol) prepared according to the general procedure C, to give after purification (eluent Et_2O/hexanes 1/99) **2b** (68 mg, 60%, E/Z = 1/1.3) as a colorless oil inseparable mixture of *E*/*Z* isomers.

*(S,Z)-N,N-dibenzyloctadec-3-en-2-amine (**2c**)*

The general procedure A was applied to **1** on a 0.35 mmol (100 mg) scale using the ylide of tetradecyltriphenylphosphonium bromide (387.5 mg, 0.7 mmol) prepared according to the general procedure C, to give after purification (eluent Et_2O/hexanes 5/95) **2c** (59 mg, 40%, *E/Z* = 1/20) as a colorless oil. $[\alpha]^{25}_D$ = +12.4 (*c*, 1.13, $CHCl_3$); ^{1}H-NMR (500 MHz, $CDCl_3$): δ = 0.77 (t, *J* = 7.1 Hz, 3H), 1.04 (d, *J* = 7.1 Hz, 4H), 1.06-1.19 (m, 23H), 1.63-1.76 (m, 2H), 3.31 (AB System, *J* = 14.0 Hz, 2H), 3.46 (ABX System, *J* = 13.7, 6.8 Hz, 1H), 3.62 (AB System, *J* = 14.0 Hz, 2H), 5.29-5.44 (m, 2H), 7.07-7.27 (m, 10H) ppm. ^{13}C-NMR (125 MHz, $CDCl_3$): δ = 166.9, 150.5, 139.9, 128.6, 128.5, 128.3, 128.2, 126.9, 121.5, 53.8, 51.5, 14.2 ppm. HRMS (ESI-TOF) (m/z) [M + H^+] = calcd for $C_{32}H_{50}N$ 448.3943, found 448.3949.

*(S,Z/E)-4-(Dibenzylamino)pent-2-enenitrile (**2d**)* [3-4]

The general procedure A was applied to **1** on a 0.35 mmol (100 mg) using (triphenylphosphoranylidene)acetonitrile (154 mg, 1.51 mmol), to give after purification (eluent Et_2O/hexanes 15/85) **2d** (100 mg, 72%, *E/Z* = 5/1) as a colorless oil. (*E* isomer) $[\alpha]^{25}_D$ = -150.2 (*c*, 1.5, $CHCl_3$).

*Ethyl (S,E)-4-(dibenzylamino)-2-methylpent-2-enoate (**2e**)*

The general procedure A was applied to **1** on a 0.35 mmol (100 mg) scale using ethyl 2-(triphenylphosphoranylidene)propanoate (187 mg, 0.51 mmol), to give after purification (eluent Et_2O/hexanes 15/85) **2e** (89.3 mg, 71%, *E/Z* = 20/1) as a colorless oil. $[\alpha]^{25}_D$ = -63.4 (*c*, 1.02, $CHCl_3$); ^{1}H-NMR (500 MHz, $CDCl_3$): δ =

1.26 (d, *J* = 16.0 Hz, 3H), 1.37 (t, *J* = 6.6 Hz, 3H), 1.70 (s, 3H), 3.25 (AB System, *J* = 14.0 Hz, 2H), 3.66-3.69 (m, 1H), 3.83 (AB System, *J* = 14.0 Hz, 2H), 4.27 (ABX System, *J* = 7.1, 1.7 Hz, 2H), 6.90 (dd, *J* = 9.6, 1.2 Hz, 1H), 7.25-7.44 (m, 10H) ppm. ^{13}C-NMR (125 MHz, $CDCl_3$): δ = 168.1, 141.8, 140.2, 128.7, 128.6, 128.3, 126.8, 60.7, 54.0, 51.6, 17.3, 14.3, 12.8 ppm. HRMS (ESI-TOF) (m/z) [M + H^+] = calcd for $C_{22}H_{28}NO_2$ 338.2120, found 338.2120.

*(S,E)-Methyl 4-(dibenzylamino)pent-2-enoate (**2f**)*

The general procedure A was applied to **1** on a 0.35 mmol (100 mg) scale using the ylide of methyl 2-(dimethylylphosphoryl)propanoate (171.0 mg, 0.51 mmol) prepared according to the general procedure D, to give after purification (eluent Et_2O/hexanes 15/85) **2f** (70 mg, 68%, *E/Z* = 1/20) as a colorless oil. $[\alpha]^{25}_D$ = -116.6 (*c*, 0.99, $CHCl_3$); ^{1}H-NMR (500 MHz, $CDCl_3$, 25 °C): δ = 1.26 (d, *J* = 6.8 Hz, 3H), 3.50 (app. quint, *J* = 6.3 Hz, 1H), 3.64 (AB System, *J* = 13.9 Hz, 4H), 3.78 (s, 3H), 5.96 (dd, *J* = 15.9, 1.3 Hz, 1H), 7.11 (dd, 15.9, 6.0 Hz, 1H), 7.24-7.42 (m, 10H) ppm. ^{13}C-NMR (125 MHz, $CDCl_3$): δ = 166.9, 150.6, 139.9, 128.6, 128.5, 128.3, 128.2, 126.9, 121.5, 51.7, 51.5, 14.2 ppm. HRMS (ESI-TOF) (m/z) [M + H^+] = calcd for $C_{20}H_{24}NO_2$ 310.1807, found 310.1811.

*(S,Z)-Ethyl 4-(dibenzylamino)pent-2-enoate (**2g**)*

The general procedure A was applied to **1** on a 0.35 mmol (100 mg) scale using the ylide of ethyl 2-(bis(2,2,2-trifluoroacetyl)phosphoryl)acetate (0.2 mL, 1.0 mmol) prepared according to the general procedure E, to give after purification (eluent Et_2O/hexanes 15/85) **2g** (100 mg, 78%, *E/Z* = 1/1.6) as a colorless oil. $[\alpha]^{25}_D$ = +99.3 (*c*, 1.28, $CHCl_3$); ^{1}H-NMR (500 MHz, $CDCl_3$): δ = 1.13 (d, *J* = 7.1 Hz, 3H), 3.37 (AB System, *J* = 14.2 Hz, 2H), 3.42 (s, 3H), 3.70 (AB System, *J* = 14.2 Hz, 2H), 4.34-4.40 (m, 1H), 5.75 (dd, *J* = 11.8, 0.6 Hz, 1H), 6.20 (dd, *J* = 11.8, 9.7 Hz, 1H), 7.07-7.26 (m, 10H) ppm. ^{13}C-NMR (125 MHz, $CDCl_3$): δ = 166.3, 149.4, 140.1, 128.5, 128.4, 128.3, 128.1, 126.7, 120.3, 54.1, 52.2, 51.0, 18.1 ppm.

*(R,E)-Ethyl 4-(dibenzylamino)-5-hydroxypent-2-enoate (**4a**)*

The general procedure B was applied to **3** on a 0.26 mmol (113 mg) scale using ethyl 2-(triphenylphosphoranylidene)acetate (141 mg, 0.41 mmol), to give after purification eluent AcEOt/hexanes 1/9) **4a** (89.3 mg, 65%, *E/Z* >20/1) as a colorless oil. $[\alpha]^{25}_D$ = -116.0 (*c*, 1.07, $CHCl_3$); ^{1}H-NMR (500 MHz, $CDCl_3$): δ = 1.37 (t, *J* = 6.9 Hz, 3H), 2.88 (br, 1H), 3.45-3.52 (m, 3H), 3.74-3.79 (m, 1H) 3.95 (AB System, *J* = 12.9 Hz, 2H), 2.80 (q, *J* = 7.1 Hz, 2H), 5.98 (d, *J* = 15.9, 1H), 7.02 (dd, *J* = 7.8 Hz, 1H), 7.29-7.40 (m, 11H) ppm. ^{13}C-NMR (125 MHz, $CDCl_3$): δ = 165.7, 142.2, 138.5, 128.9, 128.8, 128.7, 128.6, 128.5, 127.5, 125.9, 60.8,

60.4, 53.8, 14.3 ppm. HRMS (ESI-TOF) (m/z) [M + H^+] = calcd for $C_{21}H_{26}NO_3$ 339.1834, found 339.1836.

*(R,E)-Methyl 4-(dibenzylamino)-5-hydroxypent-2-enoate (**4b**)*

The general procedure B was applied to **3** on a 0.26 mmol (113 mg) scale using the ylide of methyl 2-(dimethylylphosphoryl)propanoate (180.6 mg, 0.54 mmol) prepared according to the general procedure D, to give after purification (eluent EtOAc/hexanes 10/90) **4b** (44 mg, 50%, *E/Z* > 20/1) as a colorless oil. $[\alpha]^{25}_D$ = -103.6 (*c*, 1.03, $CHCl_3$); ^{1}H-NMR (500 MHz, $CDCl_3$): δ = 2.20 (s, 3H), 3.64-3.68 (m, 1H), 3.70 (AB System, *J* = 13.8 Hz, 2H), 3.80 (AB System, *J* = 13.8, 2H), 4.42 (dd, *J* = 11.7, 5.6 Hz, 1H), 4.61 (ABX System, *J* = 5.7, 0.9 Hz, 1H), 6.16 (dd, *J* = 9.9, 1.8 Hz, 1H), 6.93 (dd, *J* = 9.9, 1H), 7.29-7.38 (m, 10H) ppm. ^{13}C-NMR (125 MHz, $CDCl_3$): δ = 166.1, 146.3, 138.7, 128.6, 128.5, 127.5, 123.1, 67.2, 54.5, 50.3 ppm. HRMS (ESI-TOF) (m/z) [M + H^+] = calcd for $C_{20}H_{24}NO_3$ 326.1756, found 326.1756.

*(R,Z)-2-(Dibenzylamino)octadec-3-en-1-ol (**4c**)*

The general procedure B was applied to **3** on a 0.26 mmol (113 mg) scale using the ylide of tetradecyltriphenylphosphonium bromide (387.5 mg, 0.7 mmol) prepared according to the general procedure C, to give after purification (eluent EtOAc/hexanes 5/95) **4c** (70.5 mg, 60%, *E/Z* = 37/63) as a colorless oil. HRMS (ESI-TOF) (m/z) [M + H^+] = calcd for $C_{32}H_{50}NO$ 464.3892, found 464.3890.

*(4R,5R,E)-Ethyl 4-(dibenzylamino)-5-hydroxyhex-2-enoate (**6a**)*

The general procedure B was applied to **5** on a 0.26 mmol (120 mg) scale using ethyl 2-(triphenylphosphoranylidene)acetate (141 mg, 0.41 mmol), to give after purification (eluent AcEOt/hexanes 10/90) **6a** (37mg, 40%, *E/Z* > 20/1) as a colorless oil. $[\alpha]^{25}_D$ = -137.2 (*c* 1.05, $CHCl_3$); ^{1}H-NMR (500 MHz, $CDCl_3$): δ = 1.03 (d, *J* = 6.1 Hz, 3H), 1.39 (t, *J* = 7.2 Hz, 3H), 2.92 (t, *J* = 9.9 Hz, 1H), 3.35 (AB System, *J* = 13.3 Hz, 2H), 3.91-3.96 (m, 1H), 4.00 (AB System, *J* = 13.3 Hz, 2H), 4.30 (q, *J* = 7.1 Hz, 2H), 5.91 (d, *J* = 15.8 Hz, 1H), 6.90 (dd, *J* = 15.7, 10.2 Hz, 1H), 7.28-7.38 (m, 10H) ppm. ^{13}C-NMR (125 MHz, $CDCl_3$): δ = 165.4, 141.8, 138.3, 128.9, 128.7, 127.5, 127.4, 66.8, 64.4, 60.8, 53.9, 19.6, 14.3 ppm. HRMS (ESI-TOF) (m/z) [M + H^+] = calcd for $C_{22}H_{28}NO_3$ 354.2069, found 354.2068.

*(4R,5R,E)-Ethyl 4-(dibenzylamino)-5-hydroxy-2-methylhex-2-enoate (**6b**)*

The general procedure B was applied to **5** on a 0.26 mmol (120 mg) scale using ethyl 2-(triphenylphosphoranylidene)propanoate (140 mg, 0.41 mmol). The

residue was purified by flash chromatography on silica gel (eluent AcEOt/hexanes 10/90), to afford **6b** (37 mg, 39%, *E/Z* > 20/1) as a colorless oil. $[\alpha]^{25}_{D}$ = -90.4 (*c* 1.14, $CHCl_3$); ^{1}H-NMR (500 MHz, $CDCl_3$): δ = 1.07 (d, *J* = 6.1 Hz, 3H), 1.39 (t, *J* = 7.1 Hz, 3H), 1.83 (d, *J* = 1.3 Hz, 3H), 3.25 (dd, *J* = 10.8, 9.7 Hz, 1H), 3.36 (AB System, *J* = 13.4 Hz, 2H), 3.87-3.93 (m, 1H), 4.03 (AB System, *J* = 13.4 Hz, 2H), 4.23 (br, 1H), 4.30 (q, *J* = 7.0 Hz, 2H), 6.82 (dd, *J* = 10.9, 1.4 Hz, 1H), 7.27-7.37 (m, 10H) ppm. ^{13}C-NMR (125 MHz, $CDCl_3$): δ = 167.4, 138.6, 134.9, 134.1, 128.8, 128.6, 127.4, 65.3, 63.7, 61.0, 54.1, 30.3, 19.2, 14.3, 13.8 ppm. HRMS (ESI-TOF) (m/z) [M + H^+] = calcd for $C_{23}H_{30}NO_3$ 368.2226, found 368.2227.

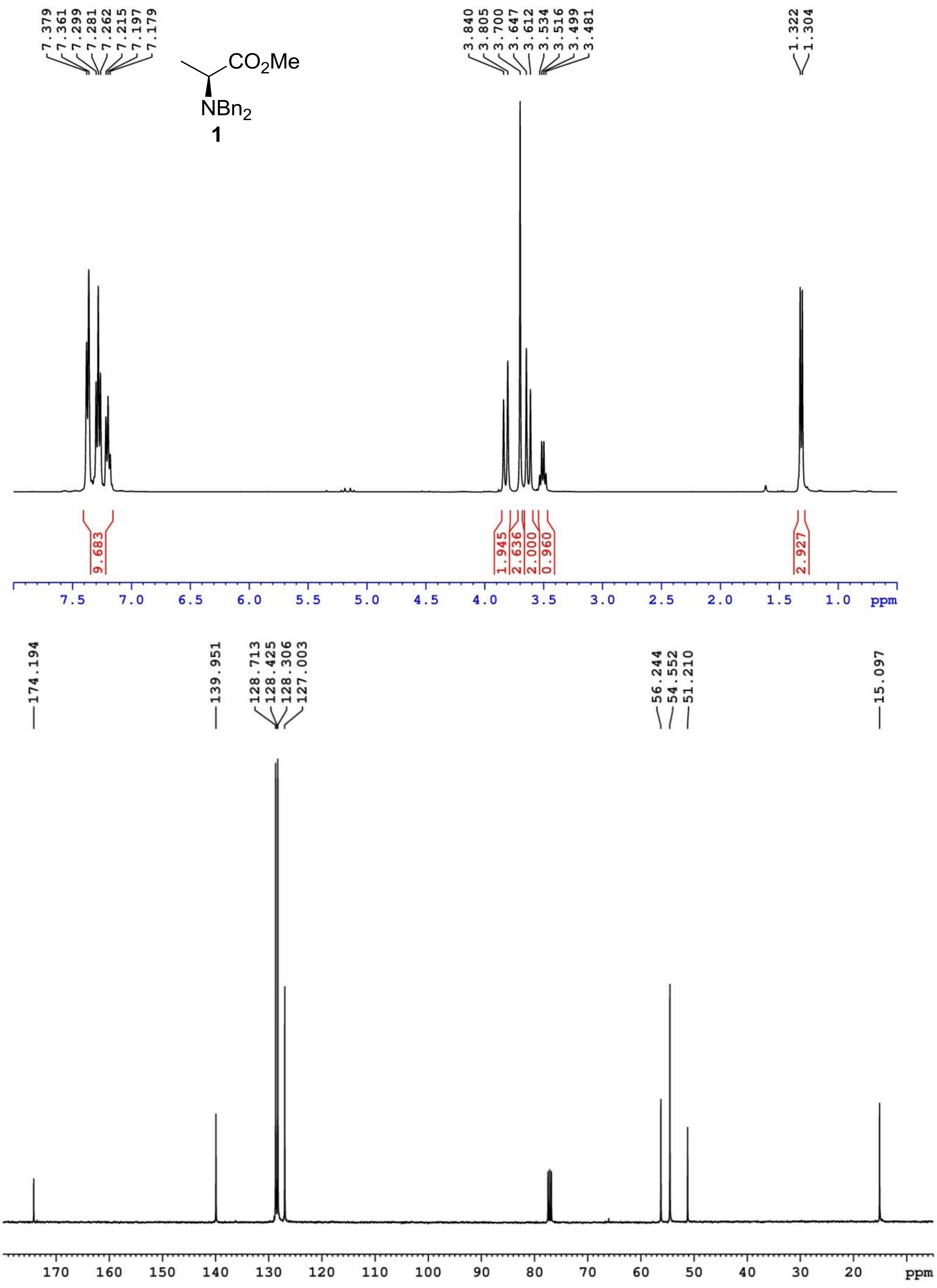

Figure S1: ^{1}H (500 MHz) and ^{13}C (125 MHz) NMR spectra of **1a** in $CDCl_3$.

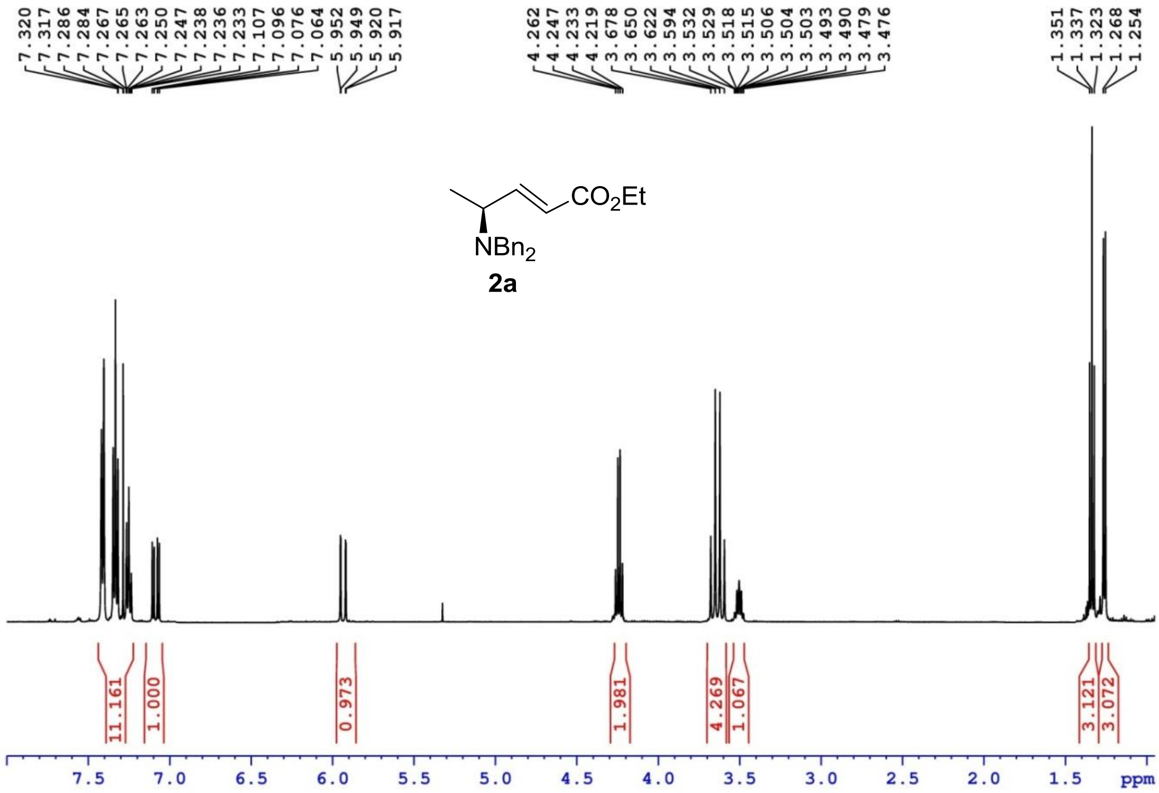

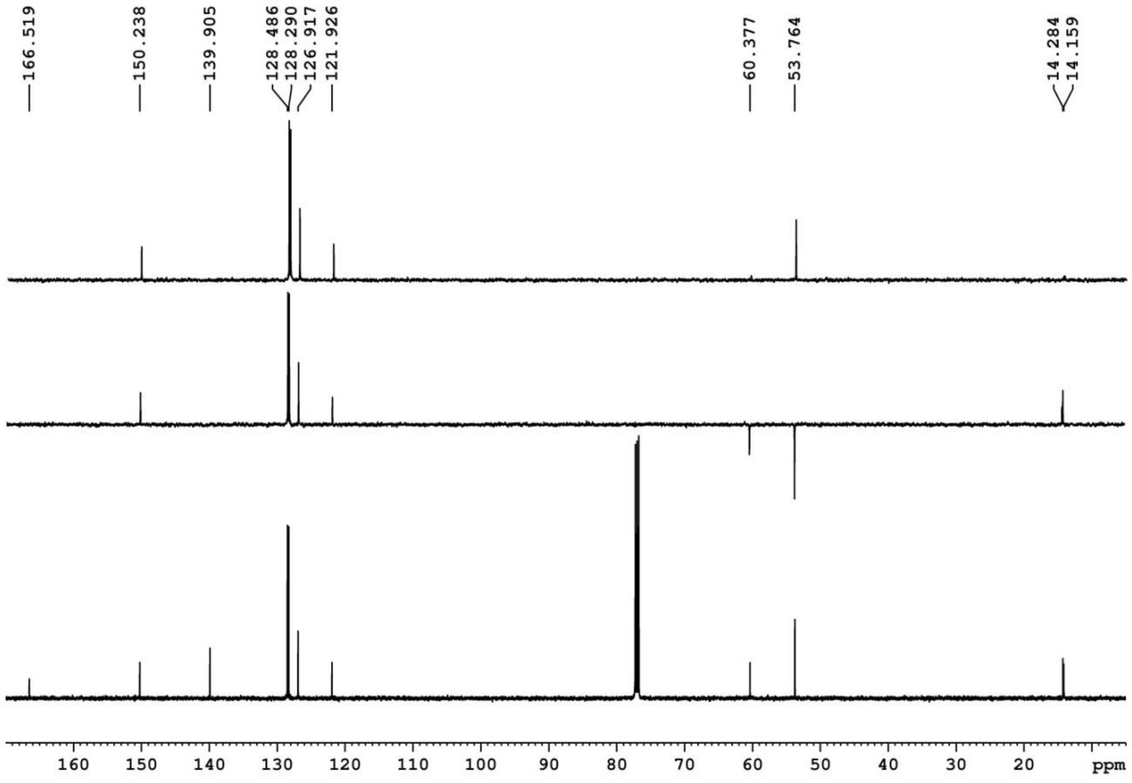

Figure S2: ^{1}H (500 MHz) and ^{13}C (125 MHz) NMR spectra of **2a** in $CDCl_3$.

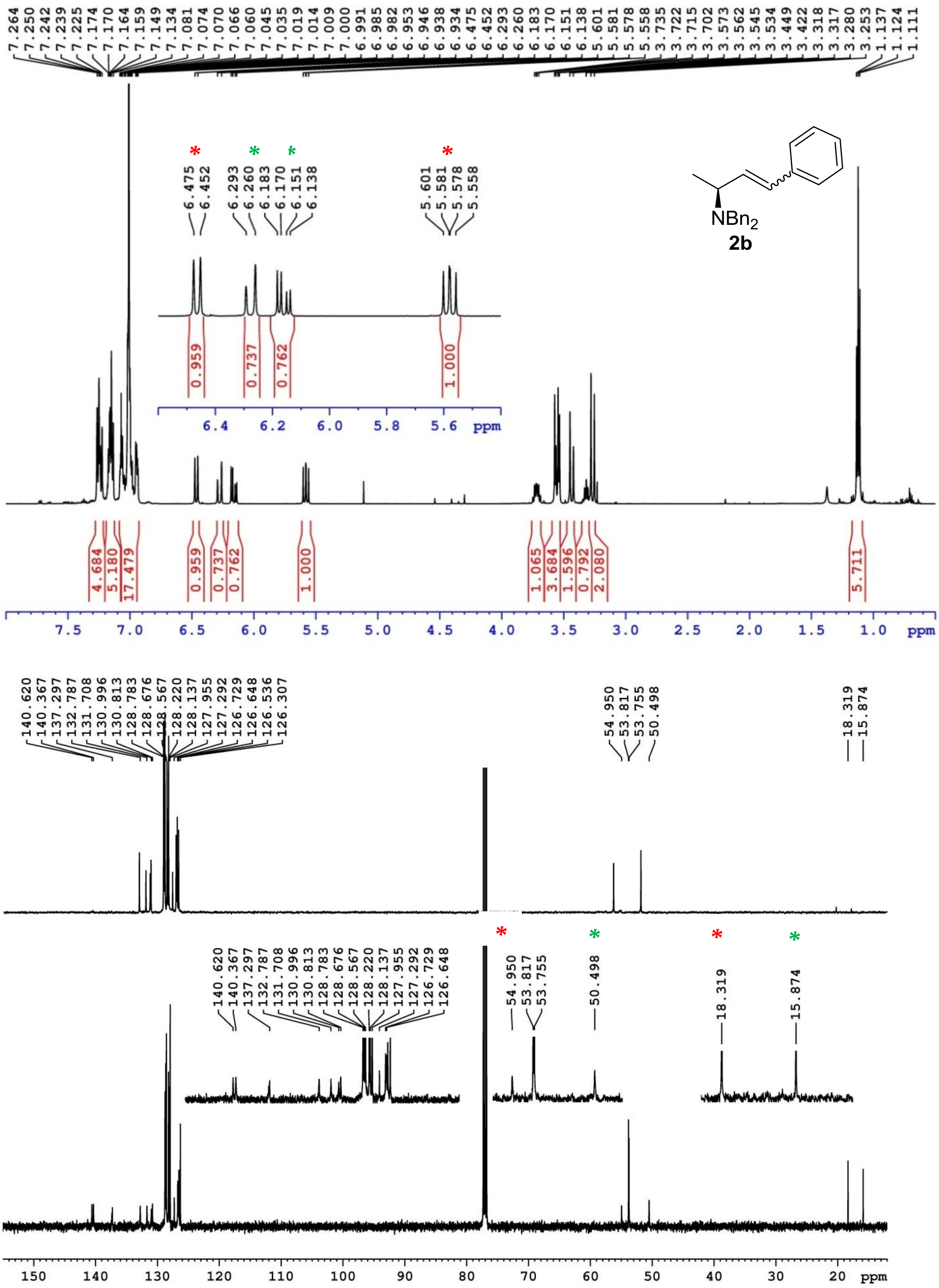

Figure S3: ^{1}H (500 MHz) and ^{13}C (125 MHz) NMR spectra of **2b** in $CDCl_3$ (* *Z* isomer, * *E* isomer) .

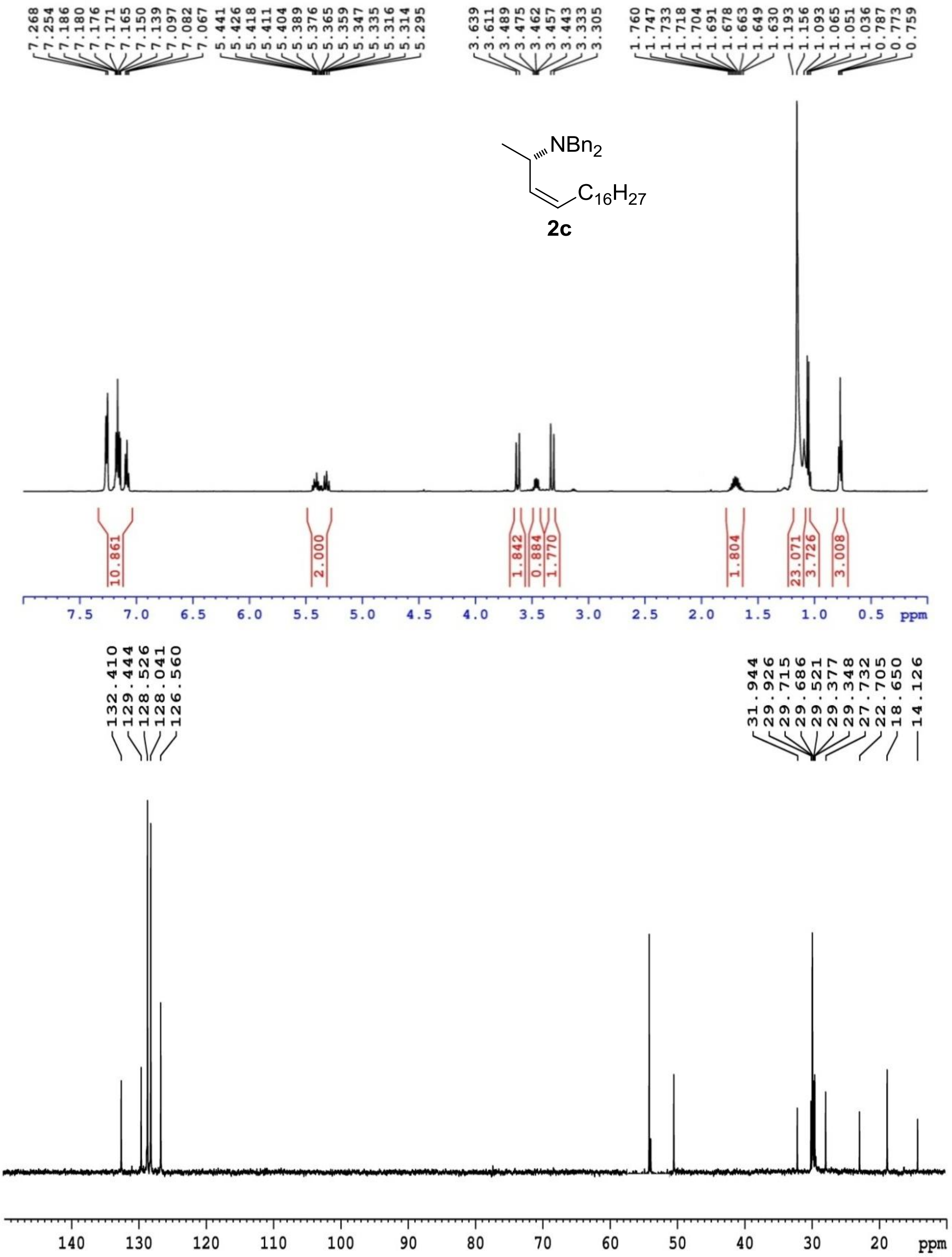

Figure S4: ^{1}H (500 MHz) and ^{13}C (125 MHz) NMR spectra of **2c** in $CDCl_3$.

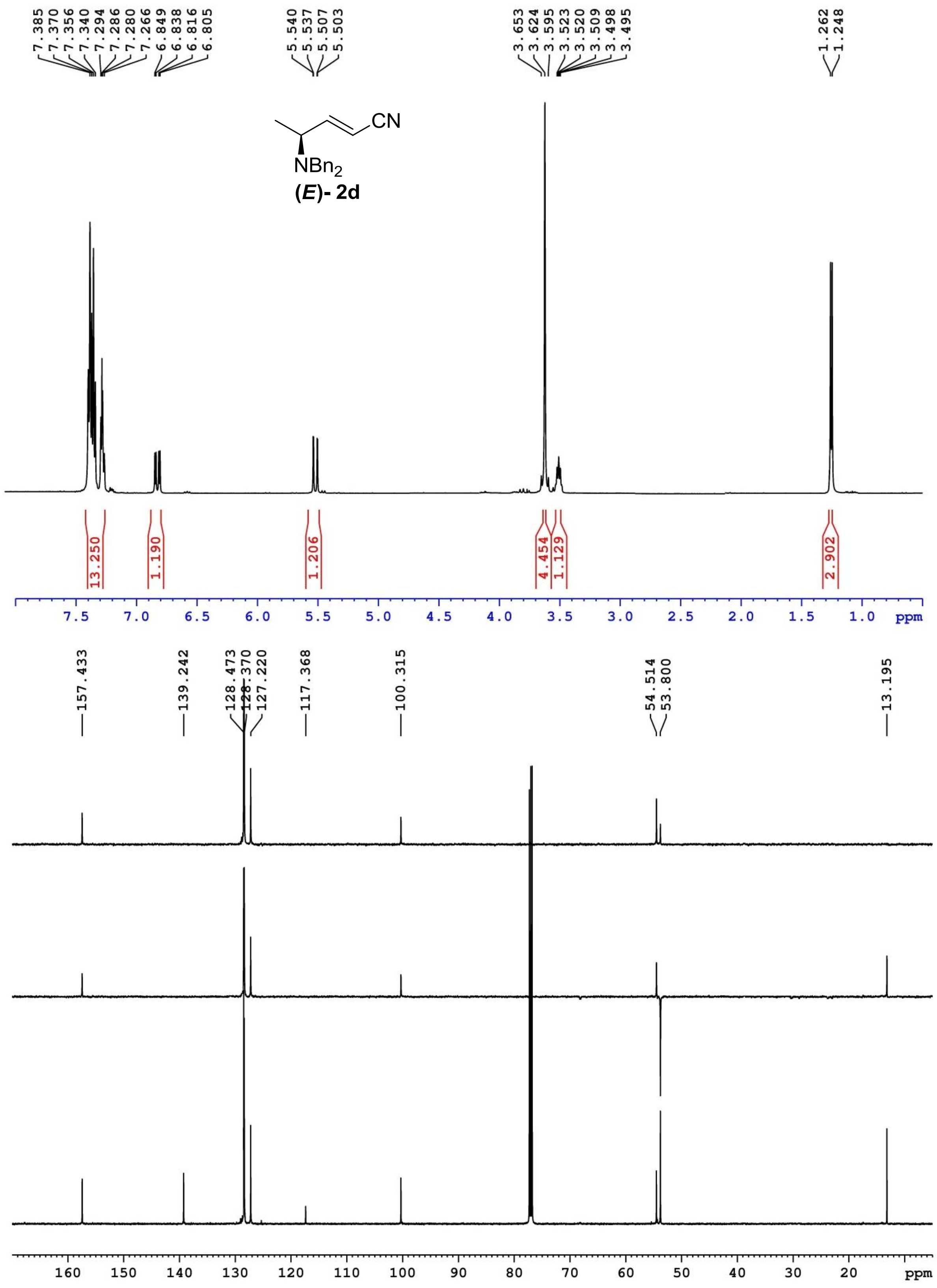

Figure S5: ^{1}H (500 MHz) and ^{13}C (125 MHz) NMR spectra of ***(E)*-2d** in $CDCl_3$.

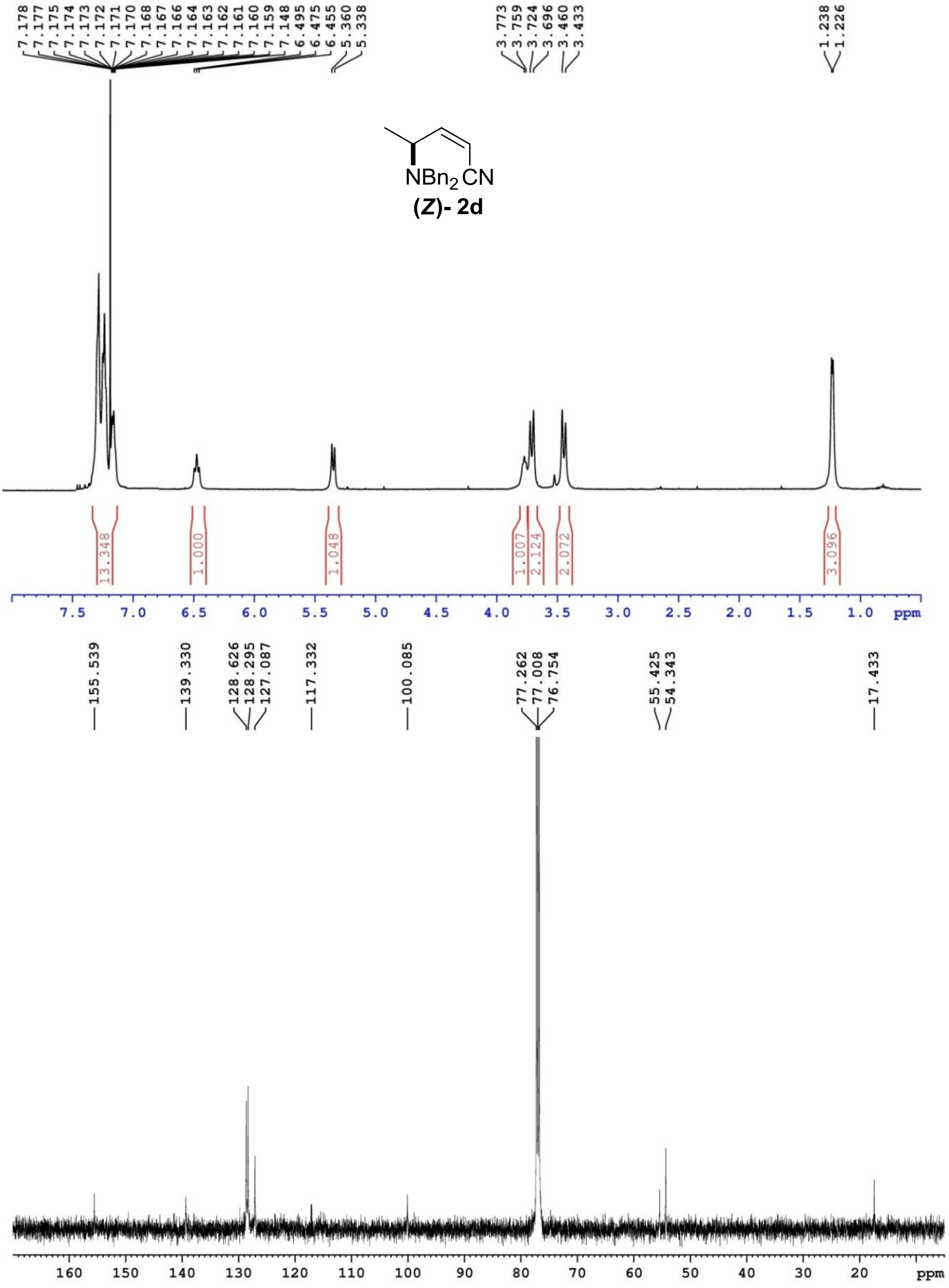

Figure S6: ^{1}H (500 MHz) and ^{13}C (125 MHz) NMR spectra of **(*Z*)-2d** in $CDCl_3$.

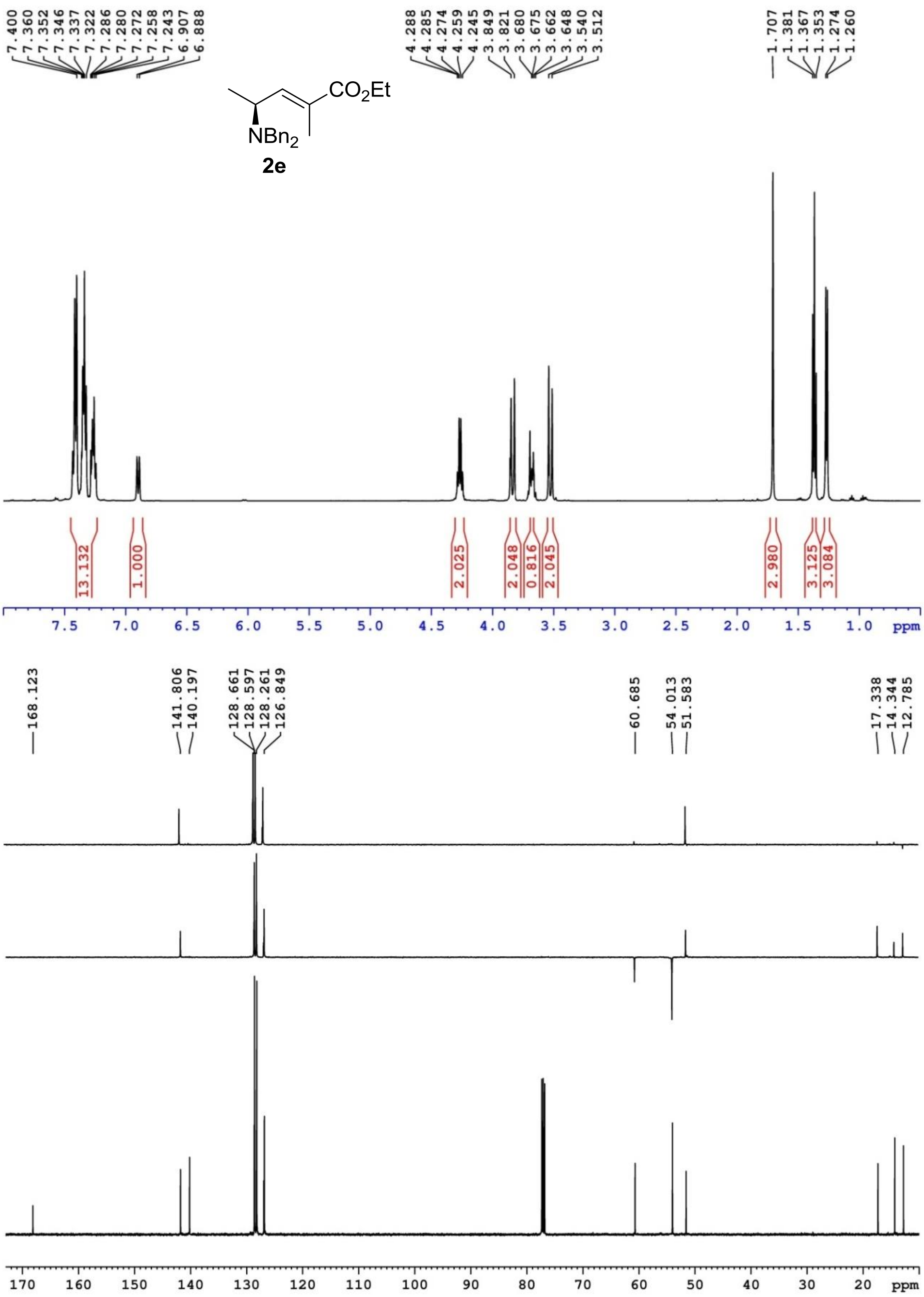

Figure S7: ^{1}H (500 MHz) and ^{13}C (125 MHz) NMR spectra of **2e** in $CDCl_3$.

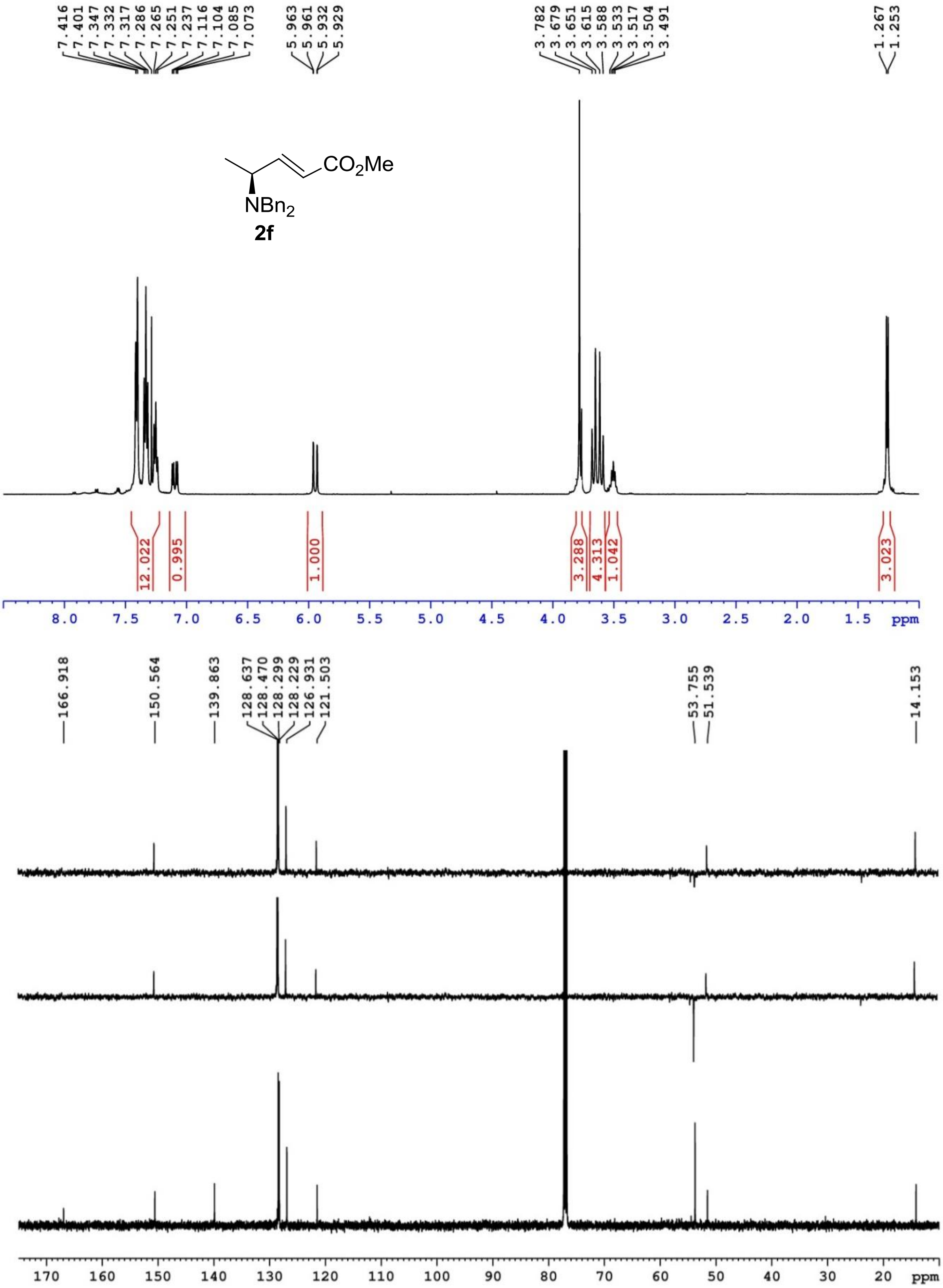

Figure S8: ^{1}H (500 MHz) and ^{13}C (125 MHz) NMR spectra of **2f** in $CDCl_3$.

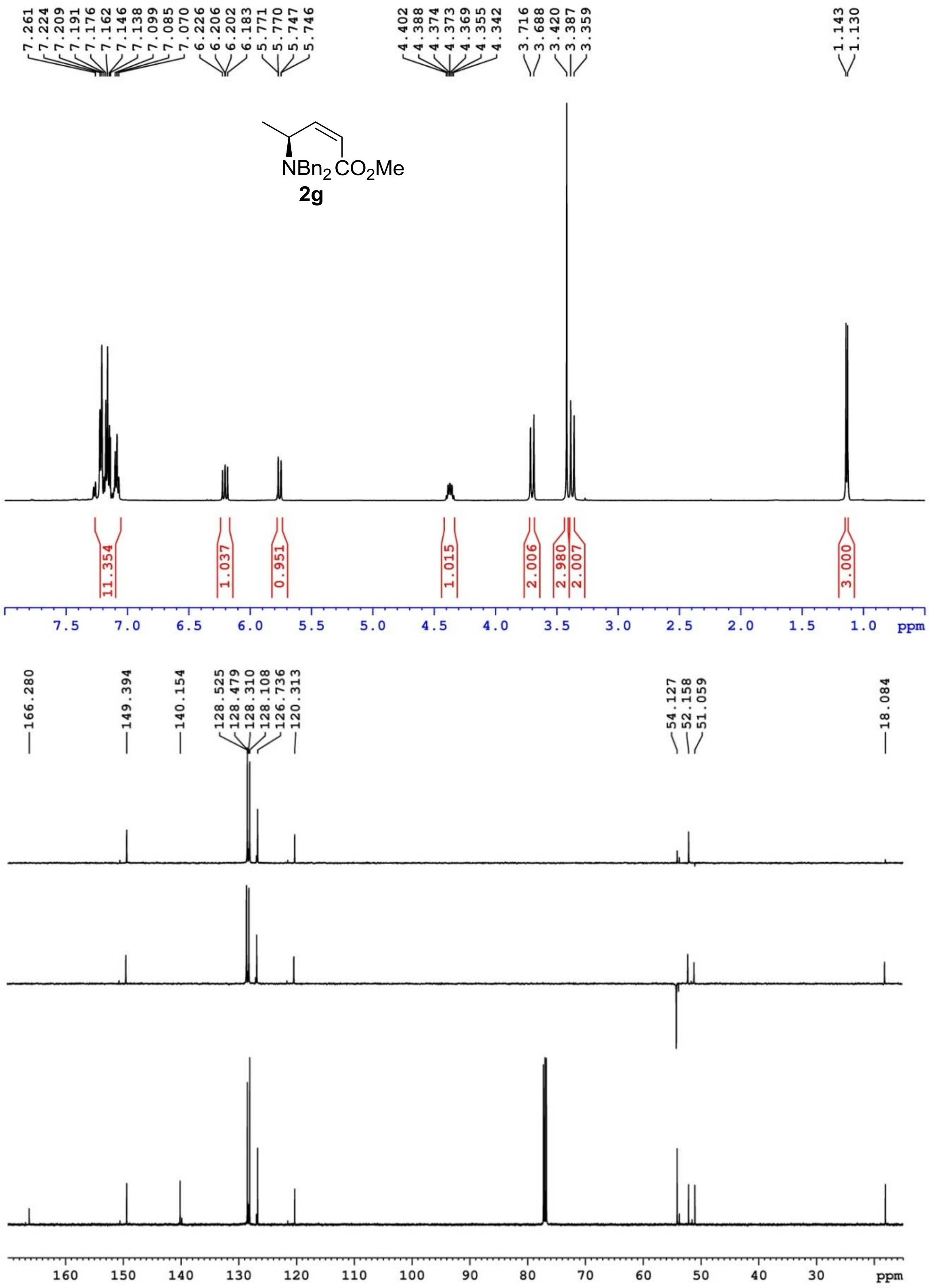

Figure S9: ^{1}H (500 MHz) and ^{13}C (125 MHz) NMR spectra of **2g** in $CDCl_3$.

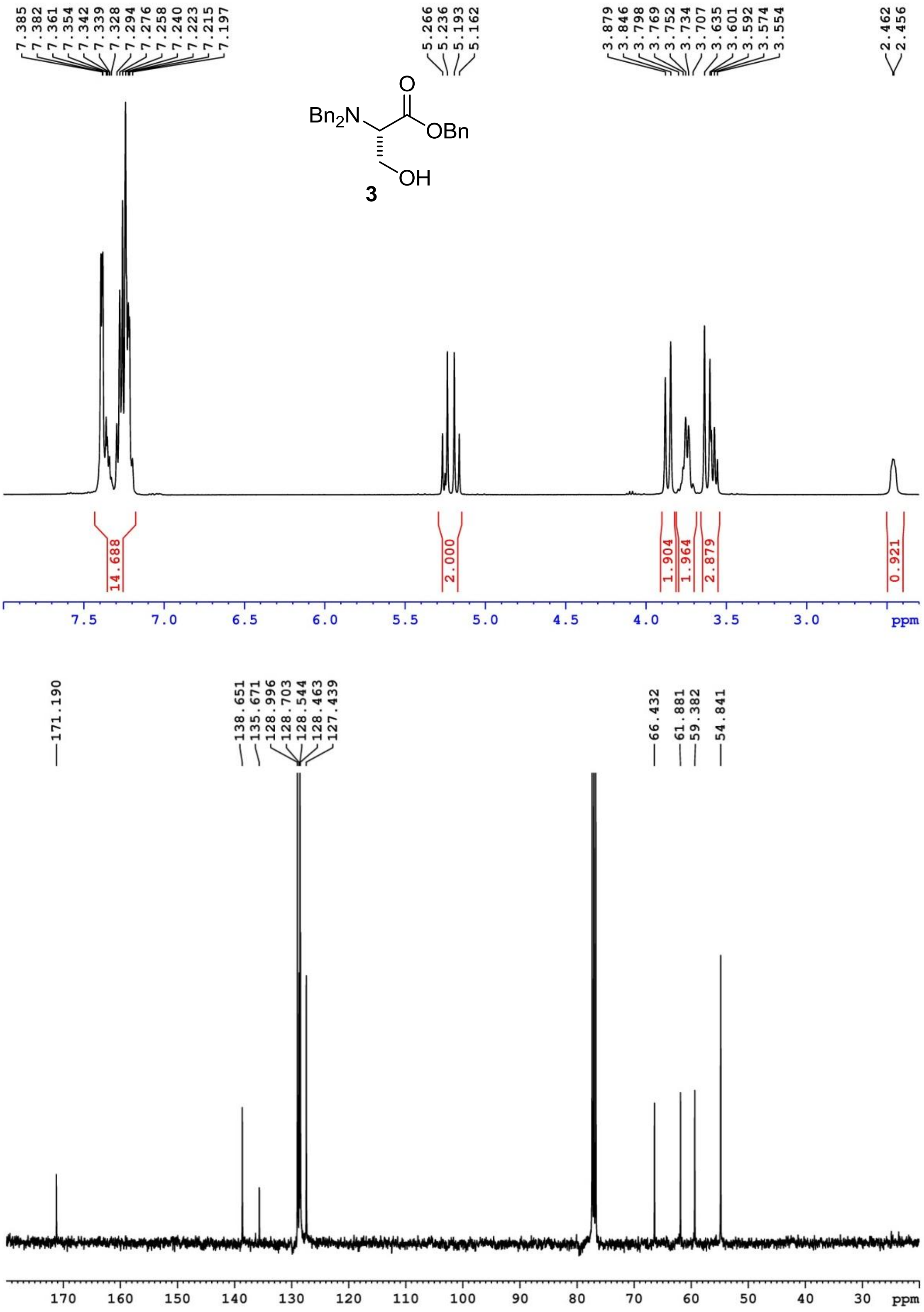

Figure S10: ^{1}H (500 MHz) and ^{13}C (125 MHz) NMR spectra of **3** in $CDCl_3$.

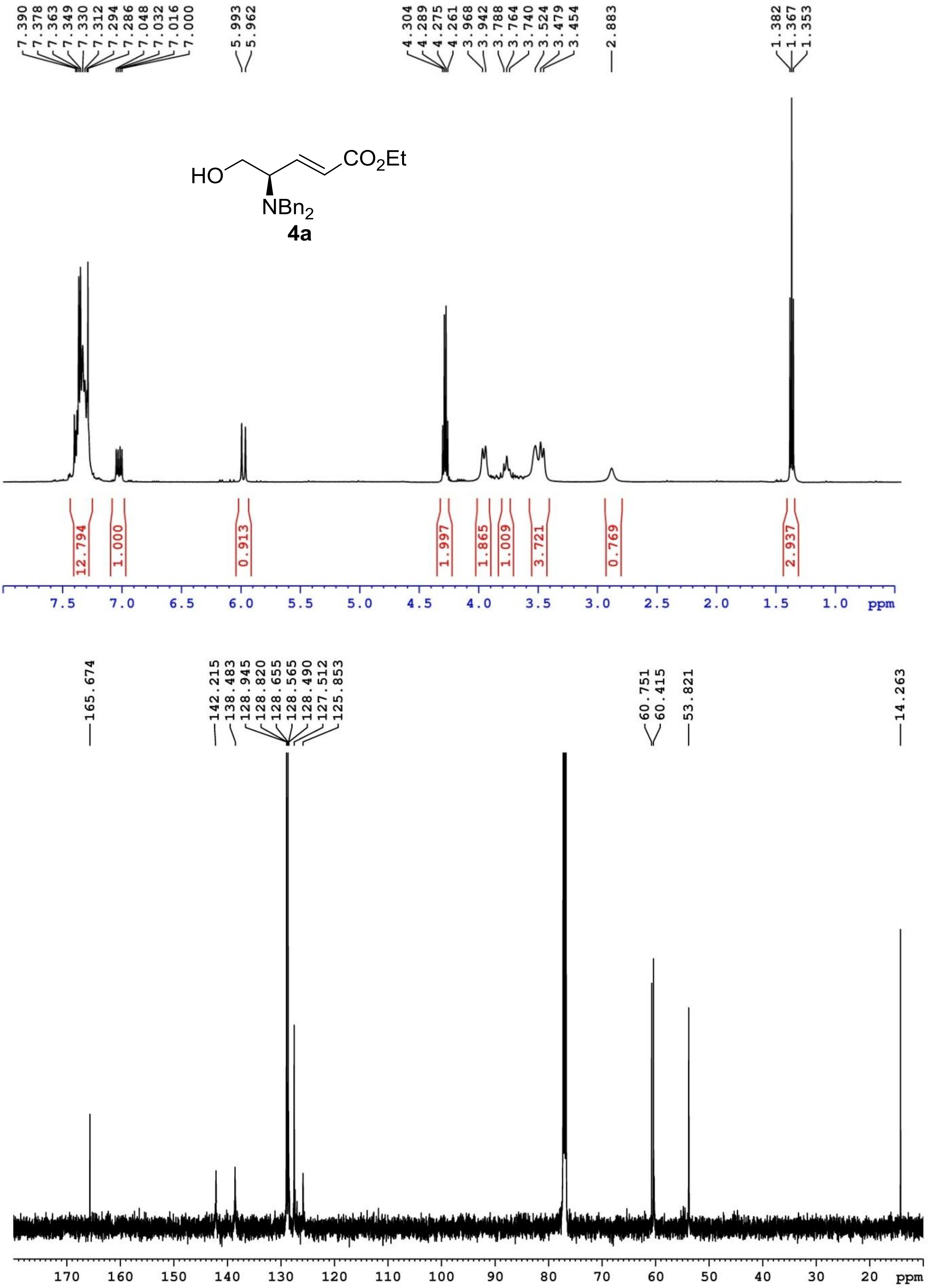

Figure S11: ^{1}H (500 MHz) and ^{13}C (125 MHz) NMR spectra of **4a** in $CDCl_3$.

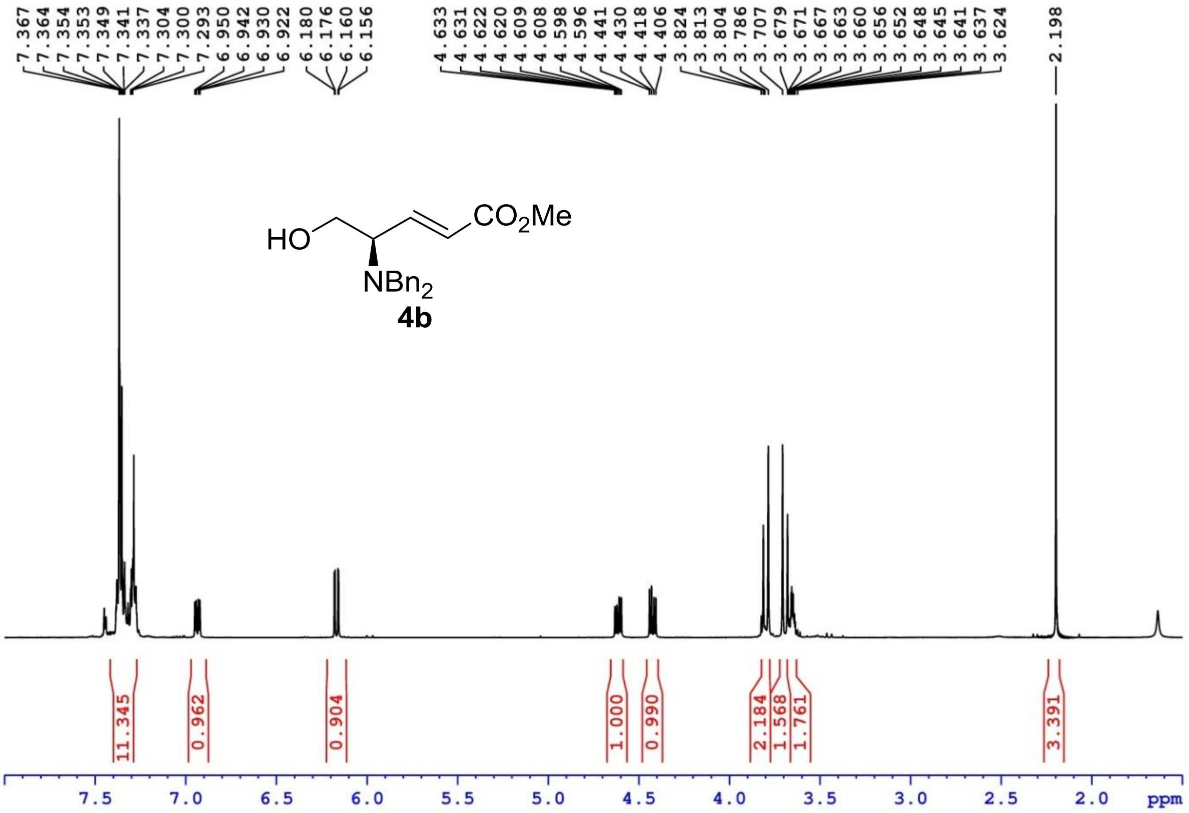

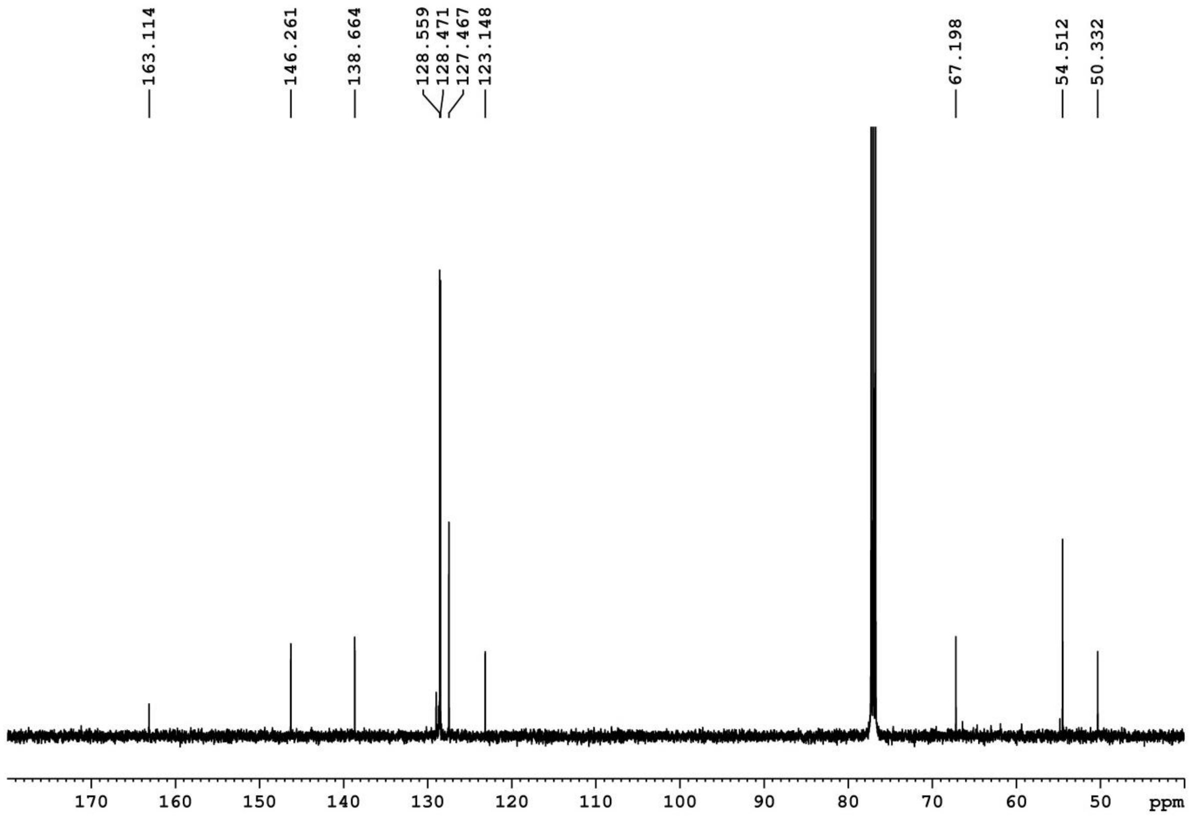

Figure S12: ^{1}H (500 MHz) and ^{13}C (125 MHz) NMR spectra of **4b** in $CDCl_3$.

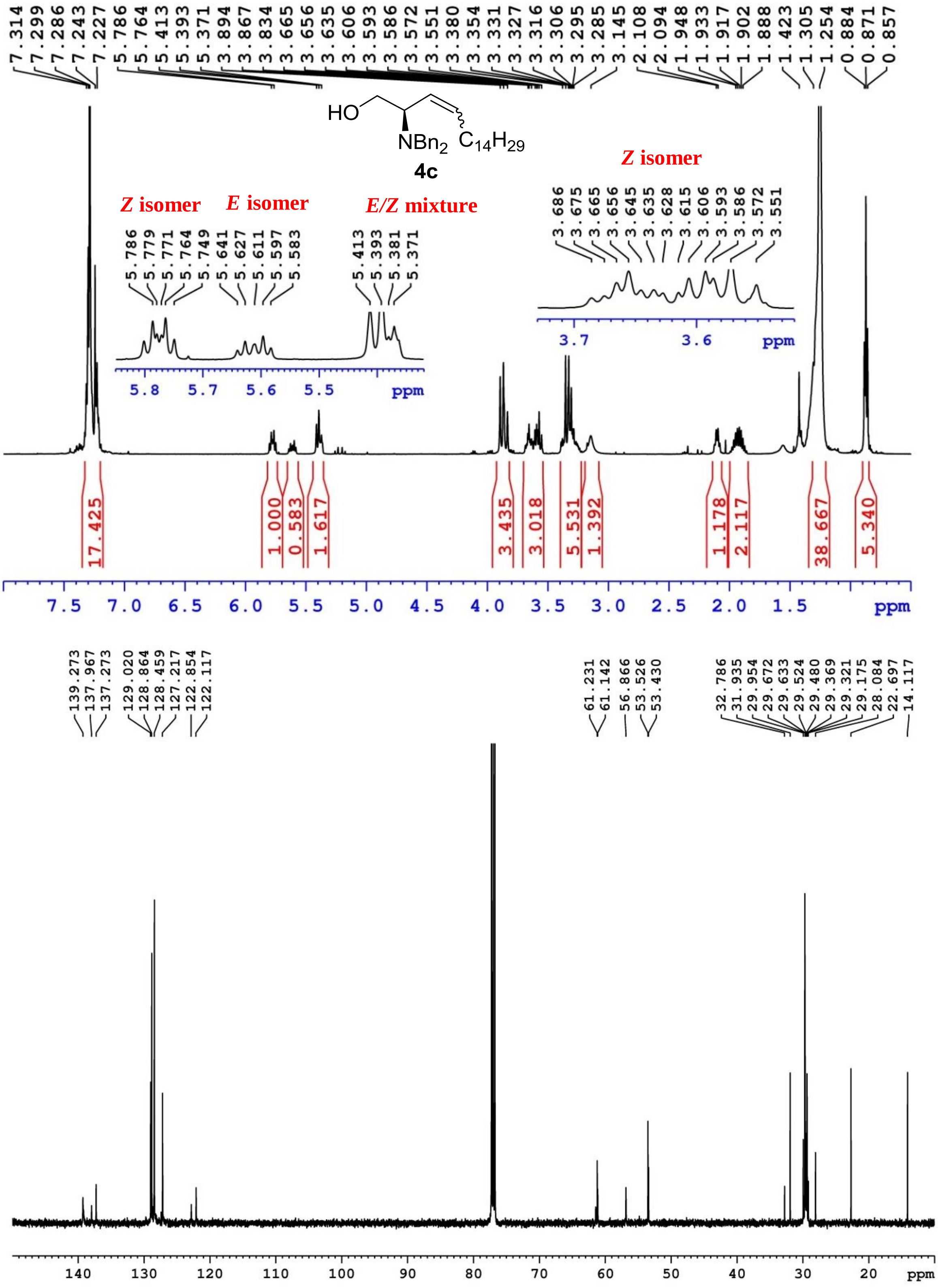

Figure S13: ^1H (500 MHz) and ^{13}C (125 MHz) NMR spectra of **4c** in $CDCl_3$.

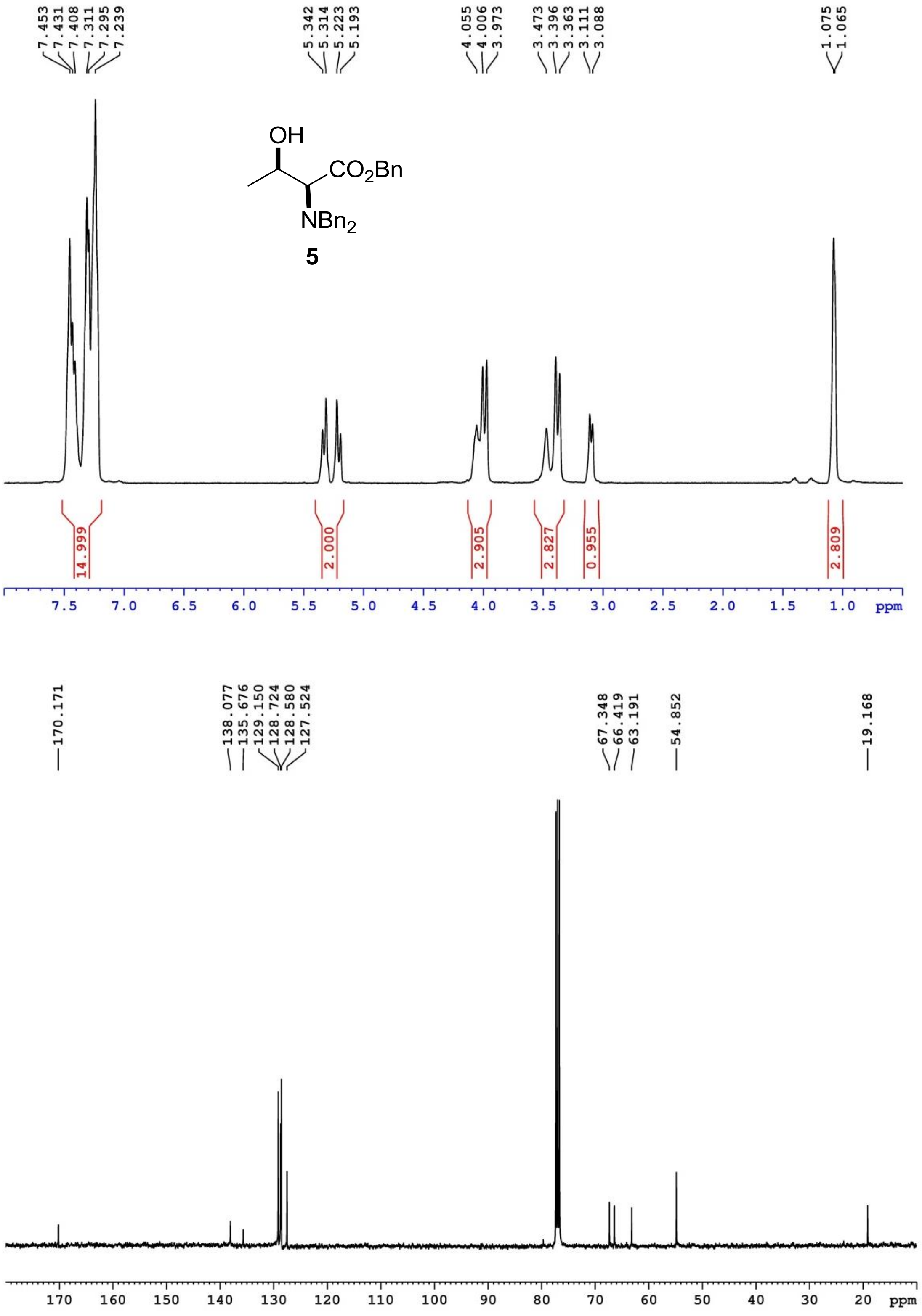

Figure S14: ^{1}H (500 MHz) and ^{13}C (125 MHz) NMR spectra of **5** in $CDCl_3$.

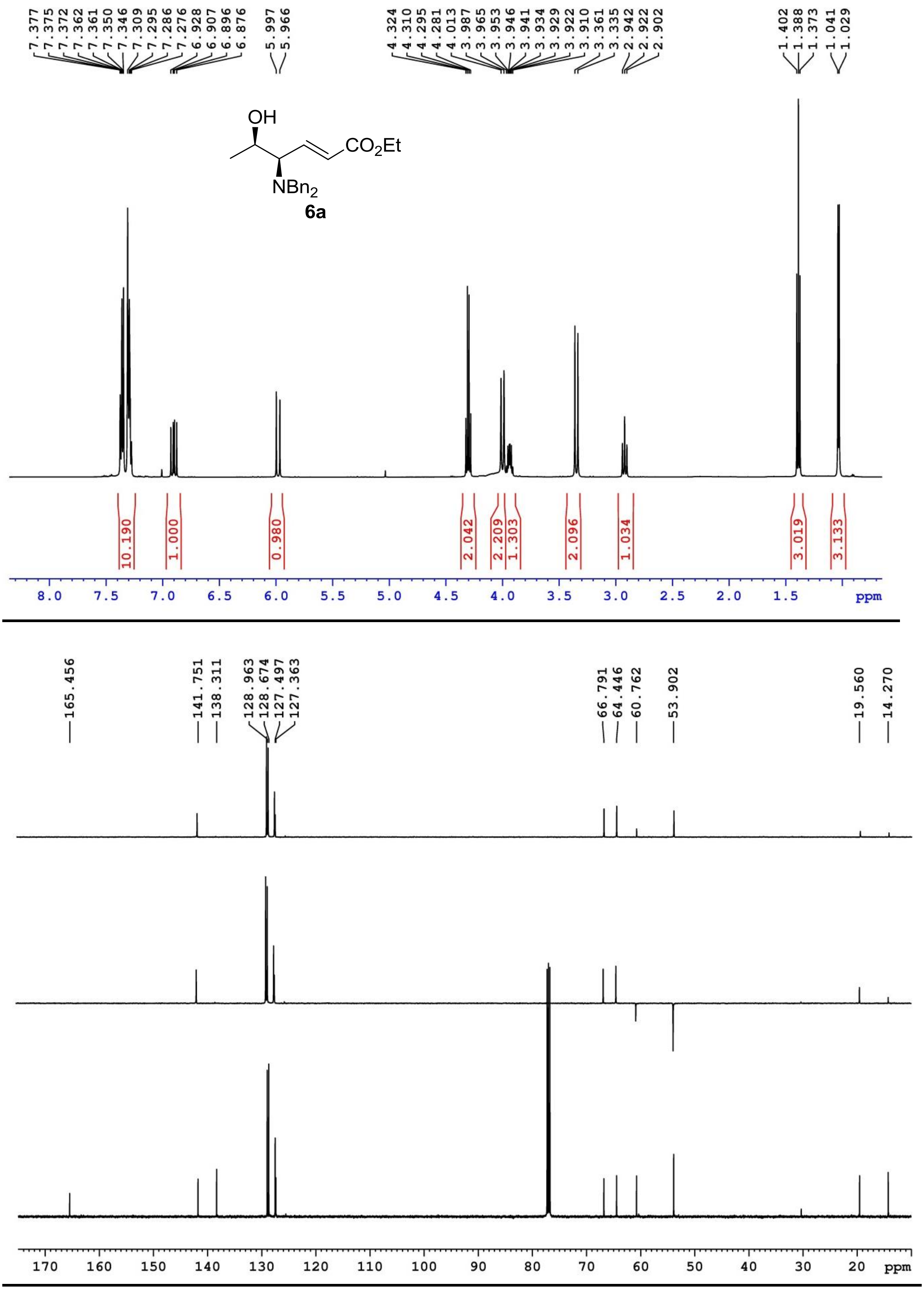

Figure S15: ^{1}H (500 MHz) and ^{13}C (125 MHz) NMR spectra of **6a** in $CDCl_3$.

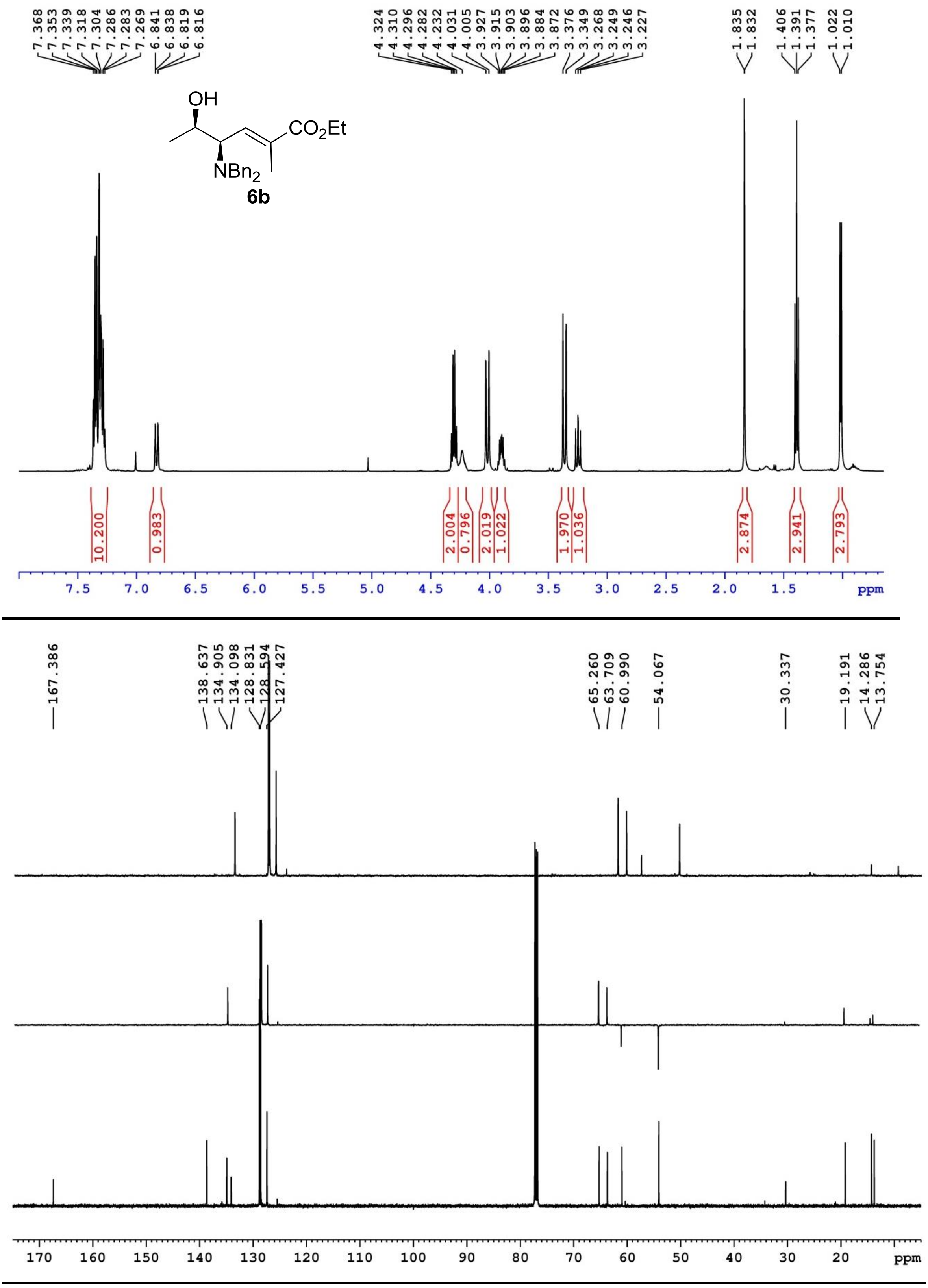

Figure S16: ^{1}H (500 MHz) and ^{13}C (125 MHz) NMR spectra of **6b** in $CDCl_3$.

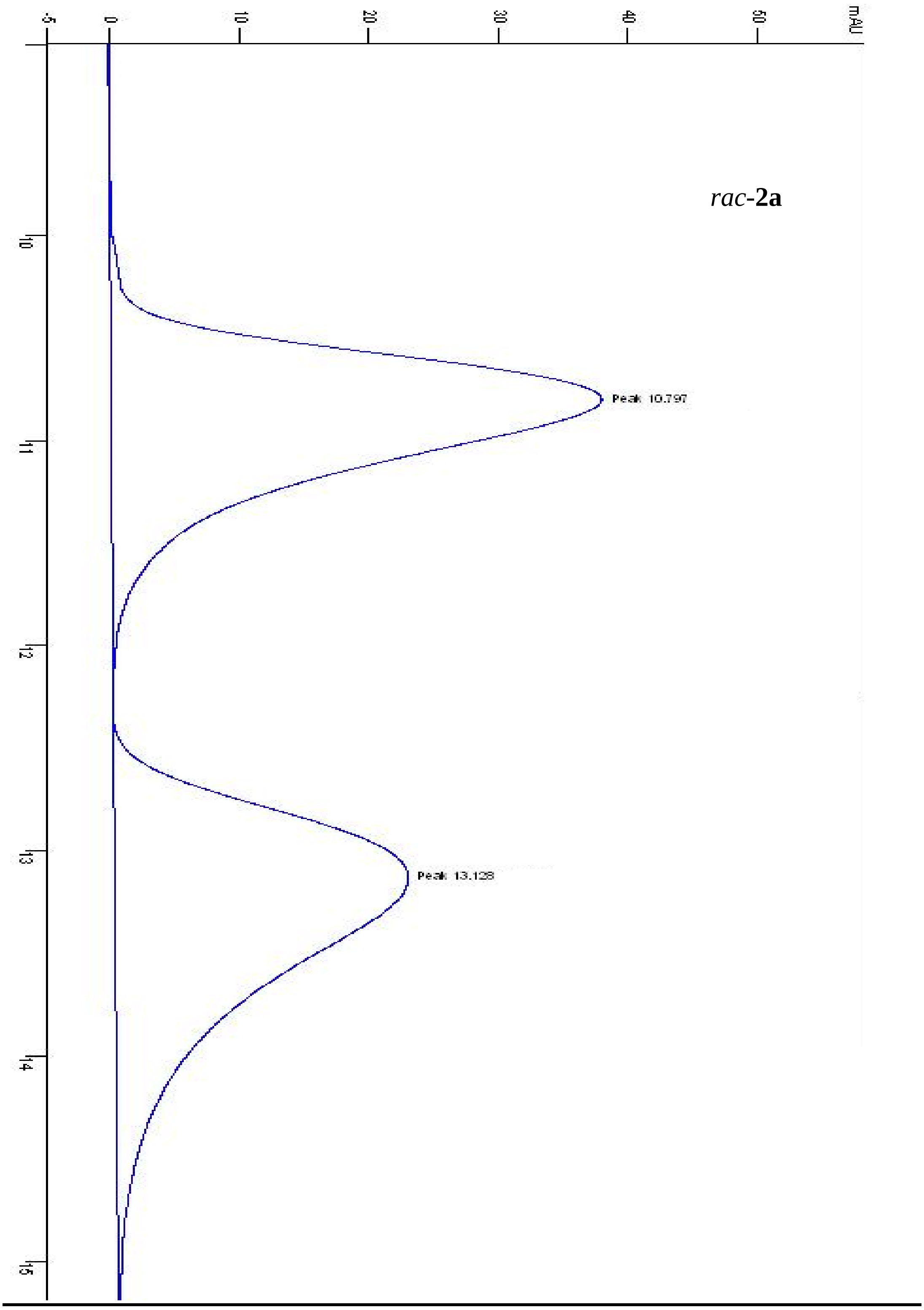

Retention time	% Area
10.79	52
13.13	48

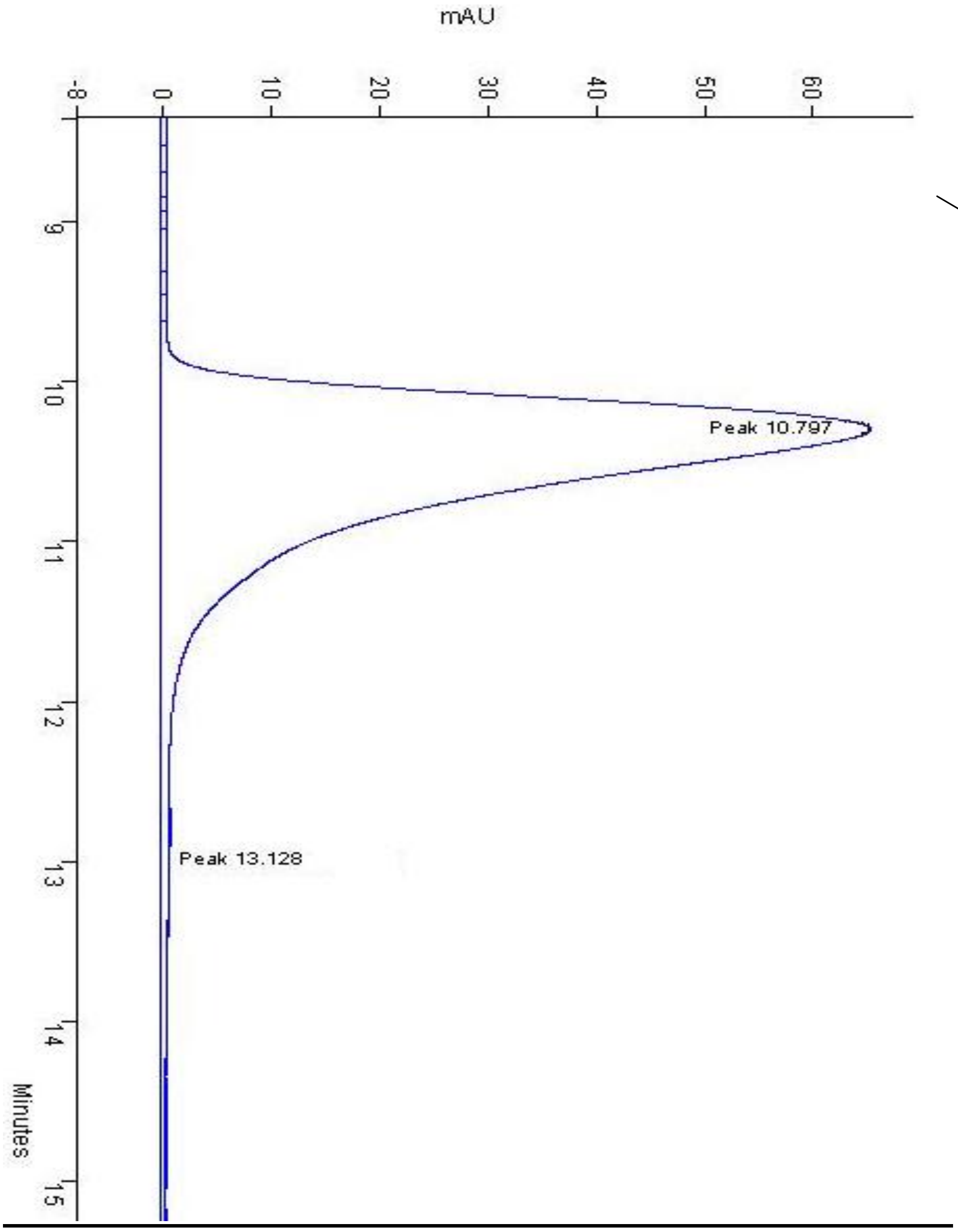

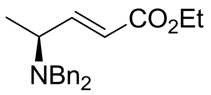

2a

Retention time	% Area
10.79	99.9
13.13	0.1

Figure S17: HPLC analysis of ***rac*-2a** and **2a**. Chiral The enantiomeric ratio was determined by HPLC analysis in comparison with racemic material (CHIRALCEL OD-H column, 95/5 *n*-hexane/2-propanol, 0.5 mL/min, major isomer: t_R = 10.79 min, enantiomer of **2a** 13.13 min. detection at 210.0 nm, 25 °C).

NMR data comparison with literature data

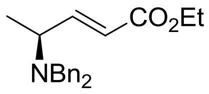

2a

Table S1: ^{1}H NMR data ($CDCl_3$) comparison with literature data for **2a**.

**This work**<br>**500 MHz**	**Ref. 1**<br>**300 MHz**
1.25 (d, *J* = 7.0Hz, 3H)	1.26 (d, *J* = 6.8 Hz, 3H)
1.34 (t, *J* = 7.0 Hz, 3H)	1.34 (t, *J* = 7.1 Hz, 3H)
3.47–3.53 (m, 1H)	3.58–3.48 (m, 1 H)
3.63 (AB system, J_{AB} = 13.9Hz, 4H)	3.60 (d, *J* = 13.9 Hz, 2H)
	3.67 (d, *J* = 13.9 Hz, 2H)
4.24 (q, *J* = 7.1 Hz, 2H)	4.24 (q, *J* = 7.1 Hz, 2 H)
5.93 (dd, *J* = 15.8, 1.6 Hz, 1H)	5.94 (dd, *J* = 15.9, 1.6 Hz, 1H)
7.09 (dd, *J* = 15.8, 6.0 Hz, 1H)	7.10 (dd, *J* = 15.9, 6.0 Hz, 1H)
7-23-7.42 (m, 10H)	7.35–7.11 (m, 10H)

Table S2: ^{13}C NMR data ($CDCl_3$) comparison with literature data for **2a**.

**This work**<br>**125 MHz**	**Ref. 1**<br>**75 MHz**
14.1	14.0
14.3	14.2
	53.6
53.8 x2	53.6
60.4	60.3
121.9	121.8
126.9	126.8
	128.0
128.3x2	128.2
128.5 x2	128.4
139.9	139.8
150.2	150.2
166.5	166.4

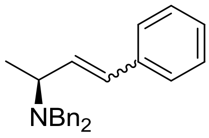

2b

Table S3: ^{1}H NMR data ($CDCl_3$) comparison with literature data for **2b**.

**This work**<br>**500 MHz (inseparable mixture)**	**Ref. 2 (*E* isomer)**<br>**200 MHz**
1.34 (d, *J* = 6.9 Hz, 3H)	1.36 (d, *J* = 6.7 Hz, 3H)
3.57-3.52 (apparent quint, *J* = 6.7 Hz 1H)	3.62-3.52 (m, 1H)
3.66 (AB system, *J* = 13.9 Hz, 2H)	3.66 (d, *J* = 13.9 Hz, 2H)
3.77 (AB system, *J* = 13.9 Hz, 2H)	3.79 (d, *J* = 13.9 Hz, 2H)
6.38 (dd, *J* = 16.1, 6.6 Hz, 1H)	6.38 (dd, *J* = 16.1, 6.3 Hz, 1 H)
6.52 (d, J = 16.1 Hz, 1H)	6.52 (d, *J* = 16.1 Hz, 1 H)
7.43-7.24 (m, 15H)	7.43-7.24 (m, 15 H)

Table S4: ^{13}C NMR data ($CDCl_{l3}$) comparison with literature data for **2b**.

**This work (*E* isomer)**<br>**125 MHz**	**This work (*Z* isomer)**<br>**125 MHz**	**Ref. 2 (*E* isomer)**<br>**50 MHz**
15.9	18.3	15.7
53.7	53.8	53.6
54.9	50.5	54.8
126.3	126.3	126.2
126.6	126.5	126.6
127.3	126.7	127.1
128.1	128.0	128.1
128.6	128.2	128.4,
128.7	128.8	128.7
130.9	131.0	130.9
131.7	132.8	131.6
140.4	137.3	140.5 x 2
140.6	140.6	141.1

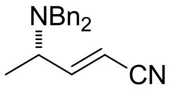

(*E*)-2d

Table S5: ^{1}H NMR data ($CDCl_3$) comparison with literature data for **(*E*)-2d**.

**This work 500 MHz**	**Ref. 3 300 MHz**	**Ref. 4 300 MHz**
1.25 (d, *J*=6.9 Hz, 3H)	1.21 (d, *J* = 6.9 Hz, 3H)	1.21 (d, *J*=6.9 Hz, 3H)
3.50 (m, 1H)	3.50 (m, 1H)	3.50 (m, 1H)
3.62 (s, 4H)	3.58 (s, 4H)	3.58 (s, 4H)
5.51 (dd, *J* = 16.4, 1.7 Hz, 1H)	5.48 (d, *J* = 16.5 Hz, 1H)	5.48 (d, *J* =16.5 Hz, 1H)
6.82 (dd, *J* = 16.4, 5.3 Hz, 1H)	6.78 (dd, *J* = 16.5, 5.3 Hz, 1H)	6.78 (dd, *J* =16.5, 5.3 Hz, 1H)
7.26-7.39 (m, 10H)	7.22-7.37 ppm (m, 10H)	7.22–7.37 (m, 10H)

Table S6: ^{13}C NMR data ($CDCl_3$) comparison with literature data for **(*E*)-2d**.

**This work 125 MHz**	**Ref. 3 75 MHz**	**Ref 4 75 MHz**
13.2	13.1	13.1
53.8	53.7	53.7
54.5	54.4	54.4
100.3	100.1	100.1
117.4	117.4	117.4
127.2	127.1	127.1
128.4	128.3	128.3
128.5	128.4	128.4
139.2	139.2	139.2
157.4	157.4	157.4

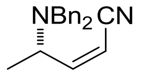

(*Z*)-2d

Table S7: ^{1}H NMR data ($CDCl_3$) comparison with literature data for **(*Z*)-2d**.

**This work 500 MHz**	**Ref. 3 300 MHz**	**Ref. 4 300 MHz**
1.23 (d, *J* = 6.7 Hz, 3H)	1.29 (d, *J* = 6.9 Hz, 3H)	1.29 (d, *J* = 6.9 Hz, 3H)
3.44 and 3.70 (2xd, *J* = 13.8 Hz, 4H)	3.52 and 3.77 (2xd, *J* = 14.4 Hz, 4H)	3.52 and 3.77 (2xd, *J* = 14.4 Hz, 4H)
3.76-3.77 (m, 1H)		
5.34 (d, *J* = 11.1 Hz, 1H),	5.41 (d, *J* = 11.2 Hz, 1H),	5.41 (d, *J* = 11.2 Hz, 1H),
6.47 (m, 1H)	6.54 (m, 1H)	6.54 (m, 1H)
7.14-7.28 (m, 10H)	7.22-7.37 (m, 10H)	7.22-7.37 (m, 10H)

Table S8: ^{13}C NMR data ($CDCl_3$) comparison with literature data for **(*Z*)-2d**.

**This work 125 MHz**	**Ref. 3 75 MHz**	**Ref. 4 75 MHz**
17.4	17.4	17.4
54.3	54.2	54.2
55.4	55.3	55.3
100.1	100.2	100.2
117.3	117.4	117.4
127.1	127.2	127.2
128.3	128.3	128.3
128.6	128.4	128.4
139.3	139.3	139.3
155.5	155.5	155.5

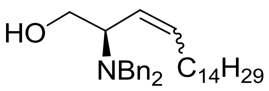

4c

Table S9: ^{1}H NMR data ($CDCl_3$) comparison with literature data for **4c**.

**This work 500 MHz (inseparable mixture)**	**Ref. 5 (*Z* isomer) 400 MHz**
0.87 (app t, *J* = 6.9 Hz, 3H)	0.90 (app t, *J* = 6.8 Hz, 3H,)
1.25-1.30 (m, 24 H)	1.19−1.45 (m, 24H)
1.86−1.99 (m, 2H)	1.87−2.04 (m, 2H)
3.14 (br, 1H)	
3.33 (dd, *J* = 10.4, 5.2 Hz, 1H)	3.33 (dd, *J* = 10.4, 5.2, Hz, 1H)
3.36 (AB system, *J* = 13.5 Hz, 2H)	3.36 (d, *J* = 13.5 Hz, 2H)
3.58 (app t, *J* = 10.4 Hz, 1H)	3.60 (app t, *J* = 10.4 Hz, 1H)
3.63−3.69 (app td, *J* = 10.1, 5.2 Hz, 1H)	3.65−3.73 (app td, *J* = 10.1, 5.2 Hz, 1H)
3.89 (AB system, *J* = 13.5 Hz, 2H))	3.91 (d, *J* = 13.5, Hz, 2H)
5.38−5.42 (m, 1H)	5.38−5.46 (m, 1H)
5.78 (app dt, *J* = 11.0, 7.5 Hz, 1H)	5.80 (app dt, *J* = 11.0, 7.5 Hz, 1H)
7.23−7.31 (m, 10H)	7.23−7.35 (m, 10H)

Table S10: ^{13}C NMR data ($CDCl_3$) comparison with literature data for **4c**.

**This work**<br>**125 MHz (inseparable mixture)**	**Ref. 5 (*Z* isomer)**<br>**100 MHz**
14.1	14.1
22.7	22.7
28.1	28.1
29.18	
29.32	
29.37	29.37
29.48	29.39
29.52	29.5
29.63	29.6
29.67	29.66
29.95	29.70
31.9	30.0
32.8	31.9
53.4	53.5
53.5	
56.8	56.8
61.1	
61.2	61.2
122.1	122.1
122.8	
127.2	127.2
128.5	128.5
128.8	128.8
129.0	
137.3	137.3
137.9	
139.2	
139.3	139.3

REFERENCES

[1] Concellón, J. M.; Méjica, C. *Eur. J. Org. Chem.* **2007**, 5250-5255.

[2] Concellón, J. M.; Suárez, J. R.; del Solar, V. *Org. Lett*. **2006**, *8*, 349-351.

[3] Baeza, A.; Casas, J.; Nájera, C.; Sansano, J. M. *J. Org. Chem.* **2006**, *71*, 3837-3848.

[4] Baeza, A.; Nájera, C.; Sansano, J. M.; Saá, J. M. *Chem. Eur. J*. **2005**, *11*, 3849-3862.

[5] Cresswell, A. J.; Davies, S. G.; Lee, J. A.; Morris, M. J.; Roberts, P. M.; Thomson, J. E. *J. Org. Chem.* **2012**, *77*, 7262−7281.
